# Evaluation of non-canonical p53 functions in DNA replication and recombination for variant classification

**DOI:** 10.1038/s41419-026-08463-0

**Published:** 2026-02-28

**Authors:** Rebecca Jansche, Benedikt Heitmeir, Ulrike Faust, Helmut Pospiech, Christian Sutter, Christian Albig, Finja Hennig, Wolfgang Janni, Rita Schmutzler, Jan Hauke, Andreas C. Joerger, Lisa Wiesmüller

**Affiliations:** 1 Department of Obstetrics and Gynecology, Ulm University, Ulm, Germany; 2 Institute of Medical Genetics and Applied Genomics, University of Tübingen, Tübingen, Germany; 3 Department of Obstetrics and Gynecology, Center for Integrated Oncology (CIO), University Hospital and Medical Faculty of Heinrich Heine University, Düsseldorf, Germany; 4 Institute of Human Genetics, Department of Human Genetics, University Hospital Heidelberg, Heidelberg, Germany; 5 Center for Hereditary Breast and Ovarian Cancer, Department of Obstetrics and Gynecology, LMU University Hospital, Munich, Germany; 6 Department of Human Genetics, Hannover Medical School, Hannover, Germany; 7 Center for Hereditary Breast and Ovarian Cancer, Center for Integrated Oncology (CIO), Medical Faculty, University Hospital Cologne, Cologne, Germany; 8 Institute of Pharmaceutical Chemistry, Goethe University, Frankfurt am Main, Germany; 9 Buchmann Institute for Molecular Life Sciences and Structural Genomics Consortium (SGC), Frankfurt am Main, Germany

**Keywords:** DNA replication, Genetic testing, Molecular modelling, Breast cancer

## Abstract

Pathogenic germline *TP53* variants predispose to diverse Li-Fraumeni syndrome (LFS) phenotypes and a broad cancer spectrum, whereby carriers of hypomorphic variants cluster in a cohort with attenuated disease onset and an overrepresentation of breast cancer (BC). Recently, functional assays have gained importance among the criteria used to predict the pathogenicity of hereditary breast and ovarian cancer (HBOC) risk-gene variants. Experimental assays scoring p53 functions in transcription and growth control have contributed to variant classification, yet a significant fraction of *TP53* variants remain of unknown significance (VUS). To understand whether non-canonical functions of p53 in the fidelity control of DNA replication may aid variant classification, we subjected 23 *TP53* VUS and 20 control variants identified in the German Consortium for HBOC (GC-HBOC) to assays that monitor nascent DNA synthesis and recombination-mediated bypass of replication barriers. Our results reveal a clear functional separation between benign (B)/likely benign (LB) and pathogenic (P)/likely pathogenic (LP) variants in recombination measurements, with B/LB variants associated with high recombination frequencies and P/LP variants with low recombination frequencies. Importantly, 8/23 VUS exhibited activities within the B/LB or P/LP ranges and therefore emerge as candidates for revised classification. Variant-specific recombination activities showed significant correlations with functional scores from four earlier studies systematically analyzing canonical p53 functions. Differently, in DNA fiber spreading assays B/LB and P/LP variants showed a more heterogeneous pattern and thus did not consistently recapitulate replication slow-down and acceleration observed in the presence and absence of p53, respectively. Structural modeling of separation-of-function (SOF) variants in transcription and recombination indicates varying effects on protein stability and the conformation of surface-exposed regions, affecting for example, the flexibility of Loop 1 (L1). Intriguingly, individual SOF variants suggest that loss-of-function (LOF) in recombination may drive BC, underscoring the predictive power of this assay for low-penetrance *TP53* variants.

## Introduction

*TP53* is the most frequently mutated gene in cancer [1, 2], affecting ~30% of all BC cases [3, 4] and ~90% of ovarian cancer (OC) patients [5]. Germline P/LP *TP53* variants cause LFS characterized by highly penetrant cancer, occurring in three age-related phases: brain tumors in childhood, sarcomas and BC during early adulthood and other epithelial cancer types at later stages [6–8]. Genetic testing of LFS as well as HBOC patients and families not meeting classic LFS criteria identified P/LP *TP53* variants predominantly of the missense type [9], whereby the widespread use of next-generation sequencing (NGS) has been accompanied by a growing number of variants of unknown significance (VUS) [10]. Two major approaches first applied to *BRCA1* and *BRCA2* are used to comprehensively interpret the probability of pathogenicity: First, multifactorial prediction models integrate direct genetic evidence and clinical data, bioinformatic prediction of splicing, and protein functionality based on structural features and evolutionary conservation [11, 12]. Second, functional assays have gained significance for classification of HBOC variants, reaching sensitivities close to 100% [13]. Significant progress was even made regarding moderate and low-penetrance variants through development of gene-specific, mostly cell-based assays [14–17]. In the case of *TP53*, these assays focused on tetramer formation [18, 19] and transcriptional activation (TA) of p53 target genes [20, 21]. More recently, *TP53* variant-specific fitness scores were calculated, reflecting transcription-dependent induction of either cell death or cell-cycle arrest and DNA-damage removal [22–26]. Such datasets were assembled in public databases, pioneered at the International Agency for Research on Cancer (IARC) [27]. Guidelines for classification of HBOC variants into P, LP, B, LB and VUS have been developed by expert panels from the ACMG/AMP [28], ENIGMA [29], and the GC-HBOC [30, 31].

Although a wealth of data exists on *TP53* variants regarding TA and downstream effects, reliance on a single biochemical activity that can genetically be separated from other genome-stabilizing functions of this multifaceted tumor suppressor might fall short [32–35]. Moreover, when annotating existing information on *TP53* missense variants in the GC-HBOC database, we noticed discrepancies between functional classifications from different reports [20, 23, 24, 26]. Accumulating evidence indicates a direct regulatory role of p53 in DNA double-strand break repair and recombination [32, 36, 37]. Recently, we and others unraveled previously unknown genome-protecting functions of human p53, altering DNA replication dynamics, particularly in stem cells undergoing self-renewal [38–42]. More specifically, p53 forms an idling complex with the specialized polymerase iota (POLɩ) at DNA replication barriers to slow down replication and to promote the safe DNA-damage tolerance (DDT) pathways of fork reversal and recombination-mediated bypass [34, 39]. Given that loss-of-function (LOF) in TA, homologous recombination and DNA replication of other high-penetrance HBOC gene products like BRCA1 have been linked to pathogenicity, we decided to evaluate non-canonical functions for *TP53* variant annotation [43, 44]. Here, we employed two cell-based assays monitoring bypass of replication barriers by a recombination reporter-based approach and the dynamics of nascent DNA synthesis by the DNA fiber spreading assay. Our results show clear discrimination between P/LP and B/LB control variants in the recombination test, whereas DNA replication dynamics showed a more heterogeneous pattern, reflecting the complexity of biochemical processes underlying this readout. Analyzing such non-canonical activities of *TP53* VUS identified in the GC-HBOC provides a refined view of pathogenicity-associated phenotypes and demonstrates the power of recombination assays to capture dysfunction caused by moderate structural changes of p53.

## Materials and methods

### Recombination measurements

Following the recommendations for functional assay development [45], we performed ≥3 independent experiments per variant. Experiments were run in a blinded fashion and in randomized batches together with three positive and three negative controls each, enabling normalization to internal references to exclude inter-experimental differences. Details are provided in Supplementary Materials and Methods.

### DNA fiber spreading assay

This assay was performed following the guidelines by Brnich et al. [45], as detailed in Supplementary Materials and Methods.

### Molecular Modeling

Structures of p53 cancer variants were modeled using AlphaFold3 [46].

Structural figures were prepared using PyMOL [47].

### Statistical analysis and graphs

Graphic presentations of data and statistical analyses were carried out using GraphPad Prism version 9 (La Jolla, CA, USA). For calculation of statistically significant differences, the Kruskal-Wallis H test was applied to the non-parametric *k*-sample comparisons. In case of statistical significance, the two-tailed Mann-Whitney U test was applied for pairwise comparisons.

Additional details are provided in Supplementary Materials and Methods.

## Results

### Identification of *TP53* VUS in the GC-HBOC

**Fig. 1:**
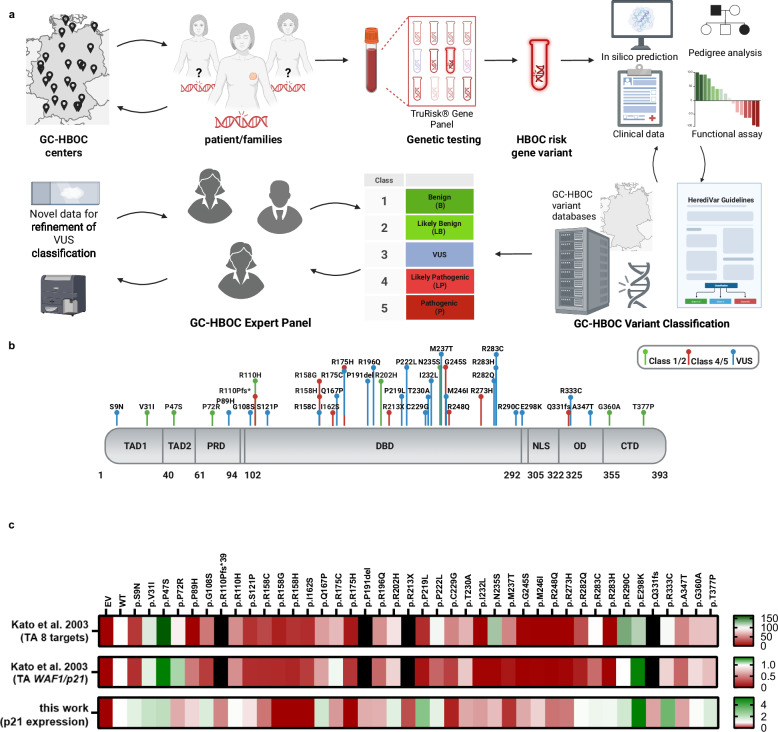
*TP53* variant identification and classification. **a** Scheme for variant detection and classification in patients and family members recruited in the GC-HBOC. Variant classification by the GC-HBOC expert panel was based on the established HerediVar guidelines [30, 31] in accordance with the recommendations of the IARC [80], the American College of Medical Genetics and Genomics/Association for Molecular Pathology (ACMG/AMP) [28] and the Evidence-based Network for the Interpretation of Germline Mutant Alleles (ENIGMA) [29, 81].The standard ACMG/AMP system has been expanded over the years with recommendations for the use of individual codes by the Sequence Variant Interpretation Working Group (SVI), and various ClinGen and non-ClinGen organizations have created gene-specific specifications (e.g. Variant Curation Expert Panels, VCEPs) such as for *TP53* [82]. Categorization into the HerediVar classes 1-5 corresponding to the IARC/ACMG/AMP classes benign (B), likely benign (LB), variants of unknown significance (VUS), likely pathogenic (LP) and pathogenic (P) was performed considering pre-existing data such as from in silico prediction tools, pedigree analyses, case-control and clinical data as well as functional assays. In February 2022, we selected 23 *TP53* variants among the fraction of 87/295 (29%) VUS in the GC-HBOC database HerediCaRe [83] for functional analysis with a high clinical need for classification. Novel data were continuously collected and considered for refined variant classification by the expert panel. **b** Scheme of the human p53 protein marking the positions of *TP53* variants analyzed in this work. VUS (blue) as well as B/LB (green) and P/LP (red) control variants are spread across the p53 domains as indicated: transactivation domain 1 (TAD1); transactivation domain 2 (TAD2); proline-rich domain (PRD); DNA-binding domain (DBD); nuclear localization signal (NLS); oligomerization domain (OD); C-terminal domain (CTD). Numbers at the bottom indicate the domain boundaries along the protein sequence. **c** Heatmap of TA of eight p53-specific promotors from Kato et al. [20], TA of *WAF1*/*p21* only [20], and p21 protein fold change from this work. WT *TP53*-specific expression of p21 is shown in white, less or more expression of p21 is shown in green and red, respectively. Black: not determined.

To evaluate the usefulness of testing non-canonical functions for *TP53* variant classification, we selected 23 VUS identified in the germline of BC patients and family members counseled at one of the GC-HBOC centers before February 2022 (Fig. 1a; Table 1). We focused on *TP53* VUS altering a single amino acid (aa) via missense or in-frame-deletion without predicted effect on RNA splicing (Fig. 1b). As controls, we selected eight B/LB and ten P/LP *TP53* variants from the same GC-HBOC cohort. The P/LP variants included truncation and missense variants; the latter comprised cancer hotspot mutations affecting protein conformation (e.g. p.R175H) or specific DNA binding (e.g. p.R273H)[48]. In Fig. 1c, heatmaps illustrate canonical functions of the investigated *TP53* variants, comparing transcriptional transactivation (TA) activities of eight p53 targets [20], of *WAF1*/*p21* separately [20], and the p21 protein expression data from our study (Supplementary Fig. 1). Reddish-colored regions in the p53 molecule reflect enrichment for LOF variants.

**Table 1:**
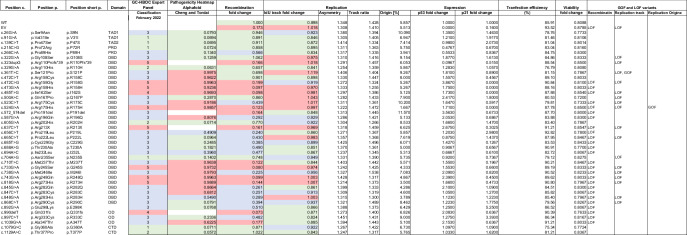
Phenotypic characterization of *TP53* variants using assays for non-canonical functions in DNA recombination and replication.

*TP53* variants, their genomic and protein alterations, their initial classification by the GC-HBOC expert panel and the functional data obtained in this study.

GC-HBOC Expert Panel variant interpretation guidelines used for classification in February 2022 were "Criteria of the German Consortium for Hereditary Breast and Ovarian Cancer for the Classification of Germline Sequence Variants in Risk Genes for Hereditary Breast and Ovarian Cancer" (Wappenschmidt 2020 GebFra, PMID: 32322110; Hauke 2021 Senologie, 10.1055/a-1342-5231).

Pathogenicity Heatmap Alphafold data (see Cheng 2023 Science, PMID: 37733863, and Tordai 2024 Sci Data, PMID: 38744964)

ambigous: 0.34-0.564

Experimental data were normalized as fold changes to the means of the following samples from the same experimental day:

Recombination: WT

Expression: WT

Viability: unperturbed cells

All values are means.

Thresholds established for functional classification in this work:

Recombination

functional: >0.65

non-functional: ≤0.20

Replication (IdU track)

functional: <0.90

non-functional: ≥0.97

LOF/GOF definition:

Recombination:

LOF: significantly lower recombination frequency compared to WT

GOF: significantly lower recombination frequency compared to EV

Replication:

track length

LOF: significantly longer tracks compared to WT

GOF: significantly longer tracks compared to EV

origin firing

LOF: significantly lower origin firing compared to WT

GOF: significantly lower origin firing compared to EV

See Supplementary Table 1 for precise *p*-values.

### Measurements of DNA recombination activities to evaluate the impact of *TP53* variants

Separation-of-function (SOF) *TP53* variants have helped distinguish non-canonical functions of p53 in DNA replication and recombination from its canonical functions in TA and growth control [34, 39]. To test whether such non-canonical functions associate with pathogenicity, we used two functional assays monitoring bypass of replication barriers [39], following the recommendations for functional assay development [45].

**Fig. 2:**
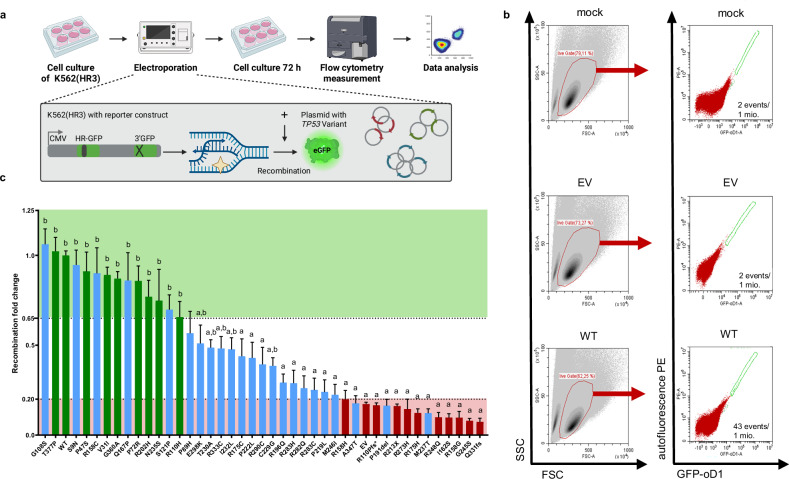
Analysis of DNA recombination as a function of the *TP53* variant. **a** Work-flow of reporter-based recombination measurements. K562(HR3) cells with genomically integrated recombination reporter were electroporated with 10 µg expression plasmid for each *TP53* variant. Cells were analyzed 72 h after electroporation using fluorescence-activated cell sorting (FACS) analysis. **b** FACS gating for recombination measurements. One million (mio.) living cells were first selected in the side scatter versus forward scatter (SSC/FSC) plot, followed by enumeration of EGFP+ cells among these living cells in a Coulter CytoFLEX B3-R1-V0 with APD detectors and GFP-oD1 bandpass filter using the autofluorescence PE/GFP-oD1 plot. Exemplary plots are shown for data obtained with mock- and EV-electroporated cells as well as after electroporation with *TP53* WT expression plasmid. **c** Waterfall plot summarizing recombination results. Columns represent mean values and SEM of recombination frequencies, normalized as fold changes to the means of *TP53* WT expressing samples from the same experimental day (mean WT: 3 × 10^-5^; *N* = 3-24, *n* = 8-141). Note that individual values of these measurements are not displayed here for clarity but can be found in the corresponding data presentation in Supplementary Fig. 2c. Relative recombination frequencies after expression of *TP53* VUS (blue), functional B/LB control variants (green) and non-functional P/LP control variants (red) are presented. Green and red shaded areas mark functional and non-functional data ranges, leaving the range of intermediate functionality in between (white). Statistically significant differences were calculated by use of Kruskal-Wallis H test followed by Mann-Whitney U test, two-sided. Statistically significant differences (*p* < 0.0001) of the variant-specific mean fold changes as compared to WT and EV are marked by a and b, respectively. Precise *p*-values are listed in Supplementary Table 1.

First, we determined recombination frequencies in the human, p53-negative K562(HR3) reporter cell line, established for p53-dependent recombination analysis [37, 39, 49]. To compare different *TP53* variants, cDNA-based expression plasmids were introduced into K562(HR3) cells by electroporation (Fig. 2a, b). K562 was chosen to exclude apoptosis-related effects [50], and transient expression to minimize protein level changes, as can be seen with stabilized oncogenic mutant proteins [51]. Quantification of p53 proteins in test samples demonstrated changes with on average 1.1-fold augmented wild-type (WT) level for B/LB and 2.2-fold for P/LP variant expression, excluding the truncated variant p.R110Pfs* from this calculation (Supplementary Fig. 1). Expression of transcriptional p53 target p21 was reduced to on average 30% of the WT level in samples expressing P/LP variants. B/LB variants showed an average of 1.3-fold expression.

Recombination measurements were performed by flow cytometry, monitoring the fraction of green fluorescent live cells resulting from reconstitution of wild-type *Enhanced Green Fluorescent Protein* (*EGFP*) over a cultivation period of 72 h [37, 39, 49]. Transfection efficiencies ranged from 75 to 94% (Supplementary Fig. 2a), i.e., were uniformly high for the different *TP53* variants, as was observed for viabilities of 72–82% according to SSC/FSC gating (Supplementary Fig. 2b). Strikingly, plotting recombination fold changes for each tested variant relative to the WT-specific value in a waterfall plot showed clear discrimination of B/LB variants (green) with a high number of EGFP+ events and P/LP variants (red) with low event numbers (Fig. 2c; Supplementary Fig. 2c). The majority of VUS-specific values (15/23, blue) were found in the intermediate range of >20% to ≤65% relative to the WT. However, five VUS-specific values were within the range of functional B/LB variants, and three were within the range of non-functional P/LP variants. Consistently, statistical calculations showed that all mean recombination frequencies in the range of B/LB variants ( >65%) were significantly different from the mean value for the empty vector (EV) control (b), and all means in the P/LP range ( ≤20%) from the mean for the WT (a) (Fig. 2c; Supplementary Table 1). Significant differences to both controls were found only in the intermediate range (a,b). These data reveal association of p53-mediated recombination with BC suppression. Thresholds for categorization could be defined, underscoring the power of this assay monitoring a non-canonical function to classify *TP53* VUS.

### Analysis of nascent DNA synthesis of *TP53* variants

**Fig. 3:**
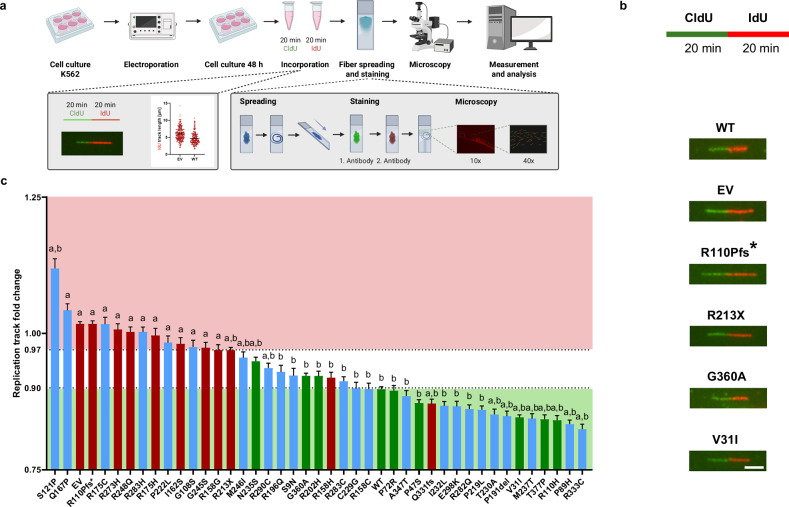
Analysis of DNA replication track lengths as a function of the *TP53* variant. **a** Work-flow of DNA fiber spreading assay. K562 were electroporated with 10 µg expression plasmid for each *TP53* variant. DNA fiber spreading assay was carried out 48 h after electroporation applying sequential 20 min pulses of incorporation of each nucleotide analogue (CldU, IdU). Fiber spreads were immunostained against CldU (green) and IdU (red), imaging performed by fluorescence microscopy and measurements of track lengths determined using Fiji/Image J. **b** Representative images of bicolored fiber tracks. Images taken from spreads after expression of the indicated *TP53* variants, after electroporation with EV as well *TP53* WT expression plasmid are shown. Scale bar represents 5 µm. **c** Waterfall plot summarizing fiber spreading assay track length measurements. Columns represent mean values and SEM of track lengths, normalized as fold changes to the means of fold change track lengths from p.R110Pfs* and p.R213X expressing control samples from the same experimental day (mean controls: 6.1 µm; *N* = 3-14, *n* = 635-6677). Note that individual values of these measurements are not displayed here for clarity but can be found in the corresponding data presentation in Supplementary Fig. 3a. Relative track lengths after expression of *TP53* VUS (blue), functional (green) and non-functional control variants (red) are shown. Statistically significant differences were calculated by use of Kruskal-Wallis H test followed by Mann-Whitney U test, two-sided. Statistically significant differences (*p* < 0.0001) of the variant-specific mean fold changes as compared to WT and EV are indicated by “a” and “b”, respectively. Precise *p*-values are listed in Supplementary Table 1.

Second, we examined whether expression of these *TP53* variants affects the speed of nascent DNA synthesis, which was previously discovered to reflect p53-mediated induction of a DDT pathway, leading to recombination at the fork [34, 39]. Therefore, we performed DNA fiber spreading assays in p53-deficient K562 cells transiently expressing the *TP53* variants analyzed in recombination (Fig. 3a). Replication speed was monitored via measurements of the track lengths of sequential 20 min pulses with thymidine analogs CldU and IdU, stained by green and red fluorescent antibodies, respectively (Fig. 3b). The waterfall plot depicting the mean fold changes of IdU track lengths relative to the means of two internal references (p.R110Pfs*, p.R213X) showed enrichment of P/LP variant-specific values in the range of long tracks, and of B/LB variant-specific values in the range of short tracks (Fig. 3c; Supplementary Fig. 3a). Thresholds for categorization were defined between the values for P/LP variant p.R213X and the WT, leaving a narrow intermediate range between <97% and ≥90% relative to the references. However, P/LP variant p.Q331fs induced a short track length in the range of B/LB, P/LP variant p.R158H and three B/LB variants (p.N235S, p.G360A, p.R202H) in the intermediate range. Reflecting the wide distribution of controls, we calculated significant differences to both EV- and WT-values (a,b) for several B/LB-specific track lengths (Fig. 3c; Supplementary Table 1). To test replication fork stalling as a possible mechanism for track shortening [52], we measured asymmetries of tricolored forks and long/short ratios of CldU and IdU tracks. Fork asymmetries did not reveal any significant changes caused by specific variants (Supplementary Fig. 3b). Track ratios showed <8% increases compared to WT, reaching significance for only four variants (p.R213X, p.M246I, p.R290C, p.C229G) (Supplementary Fig.3c; Supplementary Table 1). It has been reported that the speed of DNA synthesis is higher when origin firing is inhibited [53]. Calculating the percentages of bidirectional forks, i.e. three-colored origins which fired during the first pulse of labelling [52], we observed a highly significant change only for *TP53* p.R175H, namely a decrease (Supplementary Fig. 3d). Therefore, and in agreement with our previous work [34, 39], we do not consider fork stalling or changes in origin firing major mechanisms contributing to track shortening.

**Fig. 4:**
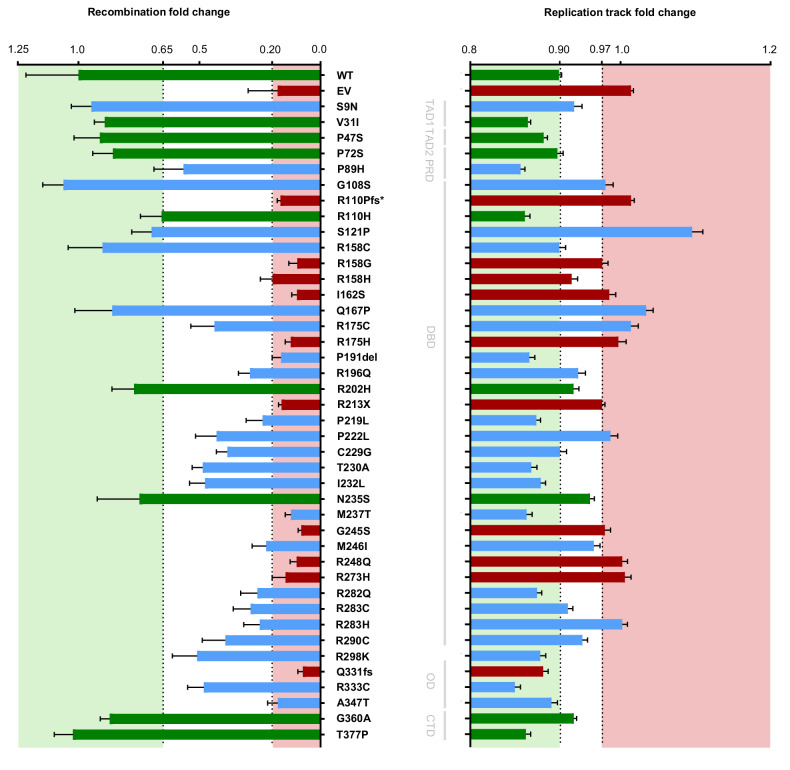
Side-by-side comparison of recombination and replication track changes. Results of recombination measurements and DNA fiber spreading assays are shown for each variant positioned along the aa sequence of the p53 protein. Columns represent mean values and SEM from the measurements shown in Fig. 2c and Fig. 3c and are colored depending on whether data are plotted for P/LP variants (red), B/LB variants (green) or VUS (blue).

Side-by-side comparison of the outcomes of the DNA recombination and fiber spreading assays showed that categorization into functional groups matches only for 21 out of 43 *TP53* variants (Fig. 4). Consistently, Spearman correlation analysis was not statistically significant (Supplementary Fig. 4). Altogether, analysis of *TP53* variants by DNA fiber spreading assays generates a more heterogeneous picture than recombination measurements.

### Recombination mediated by *TP53* variants correlates with canonical functions

**Fig. 5:**
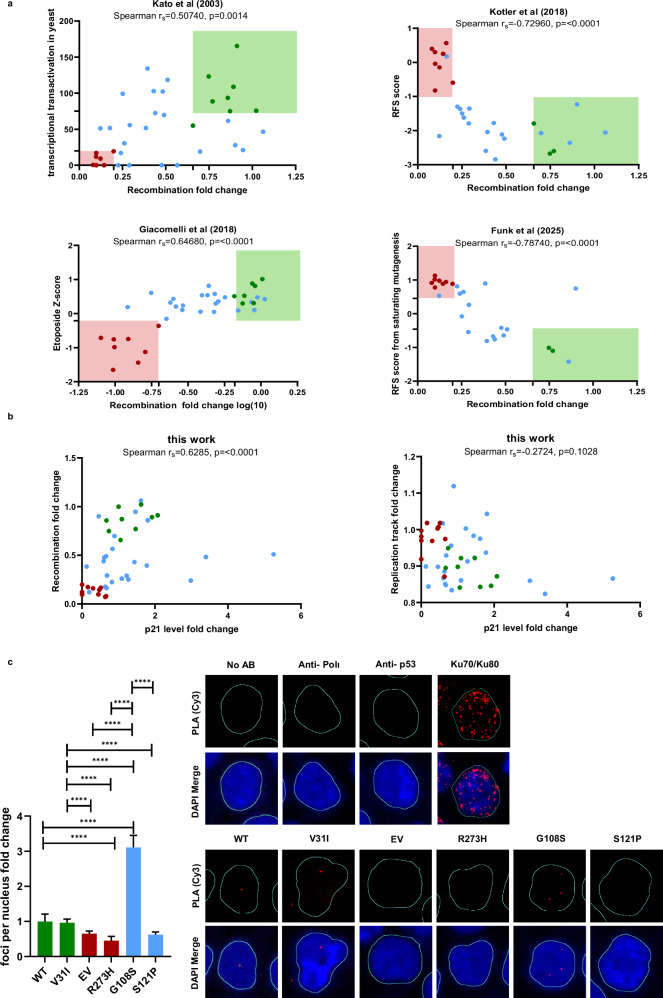
Correlations between recombination measurements and canonical p53 functions as well as impact of p.G108S and p.S121P on p53-POLɩ-complex formation. **a** Spearman correlation analyses were performed with recombination measurements listed in Table 1 and Table 2 with TA of eight p53 promotors in yeast [20], with relative fitness score (RFS) from growth of human H1299 cells [23], with etoposide Z-score from growth suppression assay in human A549 cells [24], and with RFS from saturating mutagenesis in human HCT116 cells [26]. Analysis was performed using Spearman correlation when comparing recombination values with median TA in yeast, RFS in H1299 and in HCT116. **b** Spearman correlation analyses were performed with recombination and replication measurements versus p21 protein levels, as listed in Table 1. **c** Proximity ligation assay (PLA). PLA was performed using antibodies directed against p53pSer15 and POLɩ to detect complex formation in situ. K562 cells were electroporated with EV, expression plasmid for *TP53* WT, p.R273H (P) or p.V31I (B). After culture for 48 h, cells were treated with mitomycin C (15 µM) for 45 min, recultivated in fresh medium for another 3 h and subjected to PLA. Foci numbers were normalized to the means of cells expressing WT per experiment (mean WT: 0.4 foci/nucleus). Data are presented as mean + SEM (*N* = 2–3, *n* = 318–768). Statistical significance was determined using the Kruskal-Wallis H-test followed by the two-tailed Mann-Whitney U test (*p* < 0.0001). Precise *p*-values are listed in Supplementary Table 1.

To quantitatively compare our results with functional data obtained by previously established assays for *TP53* variant classification (Table 2), we performed correlation analyses (Fig. 5a). Comparing our recombination data with TA monitored by Kato et al. [20] and allele enrichment Z-scores after etoposide treatment by Giacomelli et al. [24] revealed positive correlations, whereas relative fitness scores (RFSs) in Kotler et al. [23] and a recent study by Funk et al. [26] revealed negative correlations, reaching an r_s_ close to -0.80. When comparing the four data sets reflecting canonical p53 functions with replication track lengths, only Z-scores by Giacomelli et al. [24] showed a significant correlation (Supplementary Fig. 5a). Expectedly, mean expression levels of the transcriptional p53 target p21 in our samples significantly correlated with all four previously reported data sets (Supplementary Fig. 5b). Interestingly, our recombination but not replication data correlated with p21 protein levels (Fig. 5b). These observations confirmed the validity of recombination measurements, rather than replication track length measurements, for functional categorization of *TP53* variants, as already indicated by the clear separation of recombination frequencies specific to P/LP and B/LB variants (Fig. 2c).

**Table 2:**
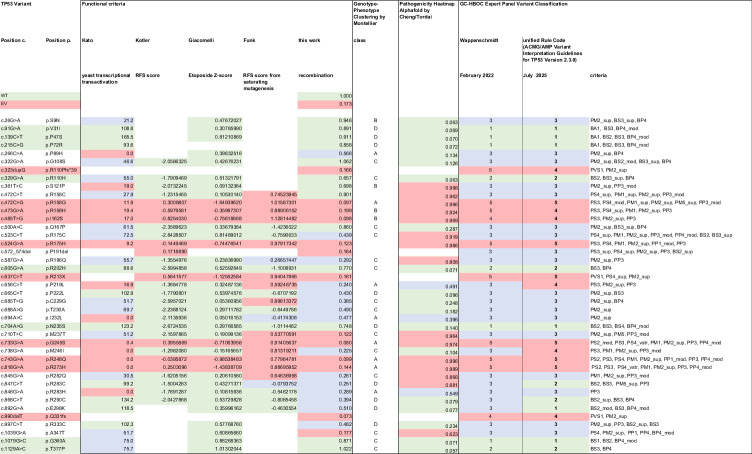
Comparison of functional *TP53* variant characterization by recombination measurements and functional assays monitoring canonical functions.

*TP53* variants, their genomic and protein alteration, their classification in functional assays and their initial and updated classification by the GC-HBOC expert panel.

**Functional classification was based on criteria from the following studies and thresholds:**

**Kato** 2003 PNAS (PMID: 12826609): transactivation of 8 p53 promoters in yeast (median)

functional: >75%

non-functional: ≤20%

partially functional: >20% and ≤75%

**Kotler** 2018 Mol Cell (PMID: 29979965): RFS score from growth of human H1299 cells (median)

LOF: ≥ -1.0

no LOF: < -1.0

**Giacomelli** 2018 Nat Genet (PMID: 30224644): Etoposide Z-score in human A549 cells (median)

LOF: ≤ -0.21

no LOF: >-0.21

**Funk** 2025 Nat Genet (PMID: 39774325): RFS score from saturating mutagenesis in human HCT116 cells (median)

benign/likely benign with 3 stars before pathogenic/likely pathogenic variants with 3 stars: -0.463055 (median)

pathogenic/likely pathogenic with 3 stars before benign/likely benign variants with 3 stars: 0.456492

Note that thresholds were defined in this work.

**this work: recombination** (mean)

functional: >0.65

non-functional: ≤0.20

**Genotype-Phenotype** ***TP53*** **variant Clustering by Montellier** 2024 iScience (PMID: 39634561): classes considering transactivation in yeast (Kato)

A: fully penetrant LFS

B: slightly less penetrant LFS

C. attenuated LFS features with lower lifetime risk

D: low lifetime risk inconsistent with LFS definitions

**Pathogenicity Heatmap Alphafold data** (see Cheng 2023 Science, PMID: 37733863, and Tordai 2024 Sci Data, PMID: 38744964)

Thresholds (August 2025):

ambiguous: 0.34-0.564

**GC-HBOC Expert Panel Variant Classification** was following

**in February 2022:** "Criteria of the German Consortium for Hereditary Breast and Ovarian Cancer for the Classification of Germline Sequence Variants in Risk Genes for Hereditary Breast and Ovarian Cancer" (Wappenschmidt 2020 GebFra, PMID: 32322110; Hauke 2021 Senologie, DOI: 10.1055/a-1342-5231).

**in July 2025**: ClinGen TP53 Expert Panel Specifications to the ACMG/AMP variant Interpretation Guidelines for TP53 Version 2.3.0 (https://cspec.genome.network/cspec/ui/svi/doc/GN009?version=2.3.0).

Selected criteria according to the ACMG/AMP Variant Interpretation Guidelines for TP53 Version 2.3.0 are indicated.

To mimic heterozygosity in the germline, we co-expressed *TP53* WT with four control variants (P: p.R110Pfs*, p.R175H, p.R273H; B: p.G360A) and the eight VUS showing functionality or LOF in recombination (Supplementary Fig. 6). When comparing recombination frequencies measured in these samples with the control lacking *TP53* co-expression (WT/EV), we did not observe dominant-negative effects (DNEs) predicted to reduce the recombination frequency below that of WT/EV (Supplementary Fig. 6a). Co-expressing p.S121P or p.G360A resulted in significantly elevated recombination compared with WT/EV (and even with WT/WT in the case of p.S121P; Supplementary Fig. 6b), supporting functionalities observed after individual expression in Fig. 2c. Interestingly, co-expressing p.M237T also increased recombination significantly, suggesting that the recombination defect seen with this variant in Fig. 2c can be rescued by WT p53. We conclude that recombination measurements detect LOF but not DNE of *TP53* variants and intriguingly, a DNE is not as representative in LFS-associated tumors as loss-of-heterozygosity and gains in copy number of the mutant allele [54].

Of further interest, we observed diametrically opposed phenotypes in recombination (functional) and replication (non-functional) for p.G108S and p.S121P (Fig. 4). When we explored their impact on p53-POLɩ complex formation via proximity ligation assay (PLA), we observed a 35% reduction in PLA foci with *TP53* p.S121P (vs. B counterpart p.V31I) and a 3.2-fold increase with p.G108S (Fig. 5c). Although both VUS were categorized functional in recombination (Fig. 2c; Table 2), we noticed that p.G108S expressing cells were at the upper, and p.S121P expressing cells at the lower limit within this functional category. Our results suggest that analysis of individual mechanistic steps of the p53-induced DDT pathway, such as PLA-based detection of p53-POLɩ complexes, may not be as strictly connected with pathogenicity as recombination, which has the potential to add another functional assay to VUS classification.

## Discussion

Our study firstly provides a systematic analysis of preclassified *TP53* variants observed in HBOC patients regarding non-canonical p53 functions in DNA replication and recombination. While canonical p53 functions guard genomic integrity indirectly via TA of target genes [25, 55], non-canonical functions ensure stability directly via safe bypass of replication barriers [39, 42, 55]. Our reporter-based recombination measurements correctly separate all P/LP and B/LB *TP53* variants, whereas measurements of replication speed by DNA fiber spreading failed to unequivocally categorize 25% of these controls. Our recombination-based analyses of 23 *TP53* VUS and 18 P/LP plus B/LB controls, which emerged in the genetic testing program of the GC-HBOC, showed highly significant correlations with the results from four systematic studies using assays for canonical p53 functions [20, 23, 24, 26]. Given that previous work demonstrated that canonical and non-canonical functions of p53 can be separated genetically [32, 39], measurements of recombination frequencies added independent functional data for *TP53* VUS classification.

The DNA fiber spreading assay is a powerful method for multiparametric analysis of different aspects of nascent DNA synthesis [52]. Here, replication speed was elevated, i.e. correctly indicated LOF, in only 9/11 P/LP/EV controls. Analysis of asymmetries and fiber track ratios excluded the possibility that deviating results in two cases (p.R158H, p.Q331fs) could be explained by slow-down of replication due to replication fork stalling. Moreover, track lengths in B/LB/WT controls were widely distributed, preventing a clear separation of functional and non-functional groups. These observations contrasted with the robust assessment of p53 functionality via recombination measurements. There are both technical and biological explanations for the inferiority of the DNA fiber assay. First, this assay involves multiple steps of experimental manipulations and therefore cannot be performed in a high-throughput format, despite careful optimization and standardization of the protocol. Differently, reporter-based recombination measurements largely rely on sensitive and specific detection of EGFP+ cells by flow cytometry. Second, recombination frequencies showed ≤14.5-fold differences, while track lengths offered a much narrower window of ≤1.4-fold differences. Third, replication can be slowed down by p53 via idling in complex with POLɩ, via HLTF- and ZRANB3-mediated fork reversal and possibly other fork remodeling mechanisms such as involving PRIMPOL and POLζ [34]. This recently refined concept of p53-POLɩ complex-dependent and –independent mechanisms is supported by our observation of diametrically opposed PLA results for p.G108S and p.S121P despite common B/LB recombination phenotype. Involvement of different fork remodelers may also explain why we found SOF variants, which appeared non-functional in replication slow-down but functional in recombination stimulation (p.G108S, p.S121P, p.Q167P) and vice versa (p.P191del, p.M237T, p.Q331fs, p.A347T) (Fig. 4).

Consequently, we focused on recombination measurement as candidate functional assay for *TP53* variant classification. When including functional data from our work in an updated standard classification of *TP53* VUS following ClinGen TP53 Expert Panel Specifications to the ACMG/AMP Variant Interpretation Guidelines for TP53 Version 2.3.0 [56], our recombination data did not support reclassifying any *TP53* VUS as LB/LP (Fig. 2c; Table 2). Most remarkably, VUS p.M237T with LOF in recombination and in Funk et al. [26] as well as p.A347T with LOF in recombination and a defect in tetramerization [18, 19] still remain VUS due to the design of the ACMG/AMP guidelines (Table 2). In particular, criterion pathogenic strong 3 (PS3) cannot be applied because the strict definition of this criterion is primarily based on the functional assay of Kato et al. [20], which showed only partial impairment but not LOF. Criterion PS3 moderate (PS3_mod) cannot be applied either, because retained functions were reported in Kotler et al. [23] and/or Giacomelli et al. [24], which generates conflicting evidence with our results and those of others [18, 19, 26]. We noticed that all VUS investigated here retained functionality in Giacomelli et al. [24] and all but p.P191del in Kotler et al. [23], whereas (partial) LOF was detectable in 17/23 VUS tested in Kato et al. [20], 9/16 VUS in Funk et al. [26], and 18/23 VUS in our recombination assay. Altogether, six variants (p.R196Q, p.P219L, p.C229G, p.I232L, p.M246I, p.R282Q) predicted to be moderately destabilized [26] and tested in all five assays showed retained functions only in the studies by Kotler [23] and Giacomelli [24]. Therefore, there is a possibility that these outgrowth assays are not sufficiently sensitive for detection of only mildly/moderately compromised p53 functions. Disadvantages of TA assays in yeast [20] are test performance at 30 ^o^C rather than at body temperature. p53 has a low intrinsic stability, a feature exploited for development of conditional mutants [57, 58], so that such experimental conditions are predicted to underestimate LOF. In support, AlphaFold Protein Structure Database Heatmap data indicate that both p.M237T and p.A347T are bona fide LP [59]. In light of these limitations, current guidelines may give too much weight to the assays described by Kato [20], Kotler [23], and Giacomelli [24]. We propose that our recombination test may serve as a valid additional assay, particularly as it monitors a non-canonical p53 function, which previously was genetically separated from canonical functions [35, 39]. All other assays analyze canonical p53 functions, which bears the risk of circularity.

Revisiting classification of *TP53* variants that show partial function in Kato et al. [20] will be of particular interest in light of recent work by Montellier et al. [8], Kasper et al. [60], and Müntnich et al. [61]. These studies refined the resolution of genotype-phenotype correlations. Kasper et al. [60] argue that *TP53* variants contribute to two clinical entities: While classical LFS associated with DNE missense variants, *TP53*-related BC rather associated with null variants. Montellier et al. [8] and Müntnich et al. [61] were able to cluster missense variants phenotypically that allowed grouping them in four classes, A-D, with A having the lowest and D having the highest TA. *TP53* variants clustering in class C were found to be associated with predisposition to BC, moderate penetrance and onset of disease around 40–50 years. Groups A and B meet classical LFS criteria with predisposition to brain, bone, soft tissue or hematological tumors, high penetrance and disease onset around 20–40 years. Class C variants often show partial function and non-deleterious Grantham biophysical prediction scores, class A and B variants show LOF. This phenotypic heterogeneity is not yet reflected in current variant interpretation guidelines for *TP53*. While P/LP controls in our study all belong to classes A and B, half of the VUS were grouped in class C (Table 2). Among class C variants, VUS p.A347T is of particular interest because it has been detected in four index patients from three BC families in the GC-HBOC, matching reports of six unrelated probands meeting revised Chompret criteria and two families meeting classic LFS criteria according to the variant curation expert panel (VCEP) [62–65]. p.A347T shows SOF between canonical and non-canonical functions, i.e., it may drive BC via recombination defect rather than via LOF in TA/growth control. Our findings suggest that recombination may serve as an independent functional assay for an updated version of variant classification guidelines.

**Fig. 6:**
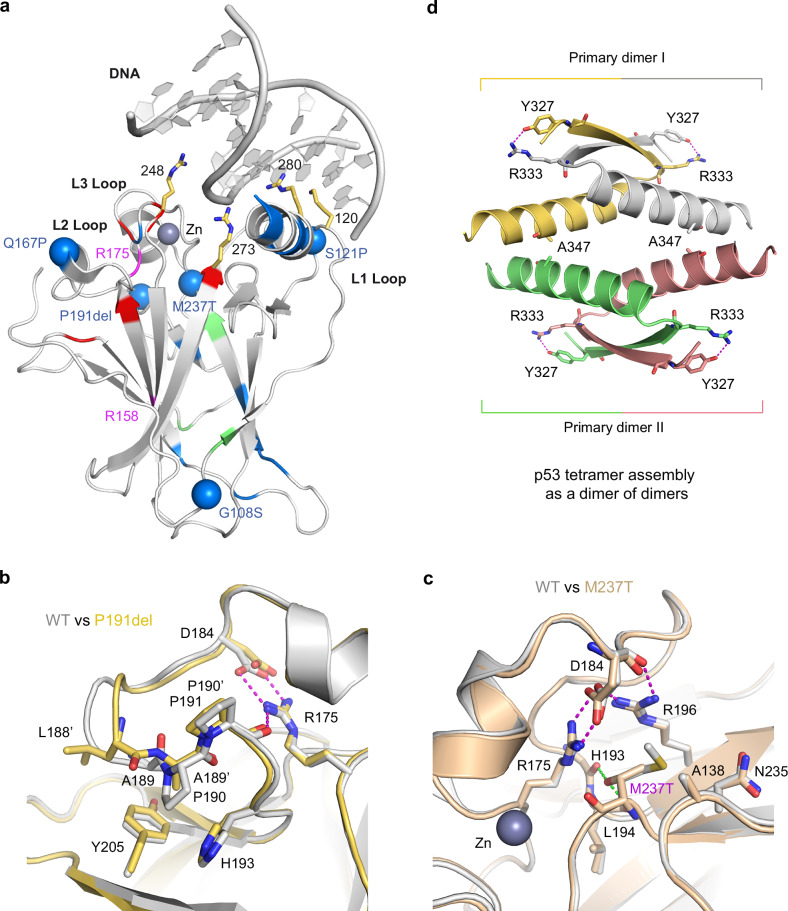
Locations and structural effects of different *TP53* variants. **a** Cartoon representation of the p53 DBD bound to DNA (PDB entry 3KMD)[84]. Side chains of key DNA-contact residues are shown as yellow stick models. The Cα atoms of the variant sites investigated in this study are highlighted in different colors: VUS in blue, B/LB in green, and P/LP in red. The two sites featuring both VUS and P/LP variants (R158 and R175) are colored magenta. The locations of selected VUS with associated SOF phenotype are highlighted with blue spheres. **b** Close-up view of the altered site in the p.P191del variant AlphaFold model (yellow) superimposed onto the WT DBD (gray; PDB entry 2XWR) [85]. **c** Close-up view of the altered site in the p.M237T variant AlphaFold model (wheat) superimposed onto the WT DBD (gray; PDB entry 2XWR). Selected hydrogen bonds seen in the variant and WT structure are highlighted with magenta dashed lines, the green dashed lines indicate hydrogen bonds mediated by the variant side chain. **d** Assembly of the p53 tetramerization domain as a dimer of dimers (PDB entry 1C26) [86]. Individual subunits are shown in different colors. Hydrogen bonds between R333 and Y327 from different subunits within the primary dimers are shown as magenta dashed lines. A347 sits at the interface between two primary dimers, which is perturbed in the p.A347T variant.

To gain further insights into the structure-function relationships underlying variant pathogenicity, we analyzed the distribution of selected variants across the structure of the DBD and their potential structural impact. Class A and B variants were reported to cluster in major structural motifs of the DBD [8]. The VUS investigated here were mostly, but not exclusively, located in solvent-exposed regions, often in loop structures (Fig. 6a). Variants p.S121P, and to a lesser extent p.G108S and p.Q167P, were functional in recombination stimulation and compromised in TA (Tables 1 and 2). These variants affect different loop regions of the DBD. p.G108S reduces the flexibility of the loop region preceding the first beta-strand, at a site distant from known functional interfaces, whereas p.Q167P alters the short helical turn within L2 on the opposite end of the DBD (Fig. 6a; Supplementary Fig. 7a). Interestingly, this site is located at the edge of the p53-53BP1 interface [66]. The p.H115N variant, which is located between p.G108S and p.S121P, was previously demonstrated to cause the opposite pattern, with LOF in p53´s intrinsic 3´-5´exonuclease activity and, consequently, idling in complex with POLɩ, replication slow-down, and recombination, while TA remained unaffected [39, 67]. Like H115, S121 is located on L1. However, S121 lies at the tip of the loop, directly adjacent to the DNA-contacting residue K120 [68, 69], which is subject to acetylation that modulates p53 binding specificity [70, 71]. The substitution of this serine with proline in p.S121P introduces rigidity into L1, thereby reducing its conformational flexibility and potentially affecting its role in DNA recognition (Fig. 6a; Supplementary Fig. 7b). Replication track lengths further reveal a GOF of p.S121P compared to p53-negative cells, which we speculate is explained by a combination of track lengthening due to reduced idling, analogous to p.H115N [39], and intermediate p21 expression unleashing PRIMPOL-mediated repriming [72]. PRIMPOL generates ssDNA gaps, which can be sealed by recombination [73], and this might explain why the p.S121P variant still shows WT-like recombination despite reduced formation of the p53-POLɩ idling complex (Fig. 5c). Intriguingly, a recent study has revealed that WT p53 can decelerate forks in two ways: either through the formation of a p53-POLɩ complex or independently of this complex but involving HLTF, ZRANB3, and PRIMPOL [34].

p.R175H is the most frequent *TP53* variant in cancer and the prime example of p53 GOF in binding novel interaction partners, activating novel pathways, inducing genomic instability, tumor initiation, promoting metastasis and drug resistance [74, 75]. Interestingly, some GOF phenotypes may depend on additional genetic alterations [26, 76]. It remains to be seen whether altered chromatin remodeling, gained interaction with MRE11, augmented aggregation with family members p63/p73 and/or itself [74, 75] are causal to the GOF of p.R175H in repressing origin firing seen here. Given that p.R175H shows severe loss of both canonical and non-canonical functions (Table 2), it is also conceivable that a synergistic defect in origin firing could arise from the loss of replication slow-down and compromised TA-dependent metabolic regulation, which normally ensures histone methylation and prevents R-loop formation [77].

The three SOF variants p.P191del, p.M237T, and p.A347T showed the opposite behavior in our functional assays, with low numbers of recombination events (non-functional) and short replication tracks (functional). The p.P191del variant shortens L2 near R175. In the wild-type structure, the backbone of P191 interacts with the guanidinium group of R175. In the deletion variant, P190 shifts into the position of P191, but this rearrangement disrupts the original hydrophobic packing interactions of P190 with H193 and Y205 (Fig. 6b). The p.M237T variant is located in L3 close to the zinc-binding site, which is critical for protein stability and positioning L3 to enable DNA binding via R248 (Fig. 6a, c). The more frequent variant p.M237I impairs both protein stability and zinc binding [78], and the p.M237T variant is also expected to reduce the conformational stability of the DBD, consistent with the reported LOF in Funk et al. [26]. All three tested variants affecting the OD (p.Q331fs, p.R333C, p.A347T) show a similar SOF pattern in replication versus recombination, highlighting the OD as an interesting domain for future studies. The p.A347T variant, for example, directly perturbs the interface between two primary dimers (Fig. 6d), preventing assembly into stable tetramers [79].

## Conclusions

Altogether, evaluating replication-associated p53 functions demonstrates a high discriminatory power of recombination measurements, with complete separation of 20 analyzed P/LP and B/LB *TP53* variants. Our analysis suggests sensitive detection of hypomorphic features associated with subtle changes of the protein structure via recombination measurements. Thus, we provide an independent assay with strong potential for the refined classification of *TP53* variants with lower penetrance predisposing to BC.

## Supplementary information

Supplementary Methods and Figures

Supplementary Table 1

Source Data

## Data Availability

The datasets generated during and/or analyzed during the current study are available from the corresponding author on reasonable request.
