# Supplementary material for: Evaluation of non-canonical p53 functions in DNA replication and recombination for variant classification
Source: Cell Death Dis. 2026 Feb 28;17(1):292. doi: 10.1038/s41419-026-08463-0 (PMC13031496; doi:10.1038/s41419-026-08463-0)
Supplement: Supplementary file 1 — Supplementary Methods and Figures [file 41419_2026_8463_MOESM1_ESM.pdf]

**SUPPLEMENTARY TEXT to**

# Evaluation of non-canonical p53 functions in DNA replication and recombination for variant classification

**Running Title:**

*TP53* variant classification by recombination data

**Authors:**

Rebecca Jansche[1], Benedikt Heitmeir[1], Ulrike Faust[2], Helmut Pospiech[3], Christian Sutter[4], Christian Albig[5], Finja Hennig[6], Wolfgang Janni[1], Rita Schmutzler[7], Jan Hauke[7], Andreas C. Joerger[8,9], Lisa Wiesmüller[1,*]

## Supplementary Materials and Methods

### Patient data and variant classification

Patients were recruited in the GC-HBOC according to the published inclusion criteria (1). Written informed consent was obtained from all individuals, and ethical approval was granted by the ethics committee of the University Clinic Cologne, Germany (#19-1360_4 “HerediCaRe”). Multigene panel analysis using genomic DNA from peripheral blood leukocyte samples was performed as described (2, 1).

### Cell Culture

Parental K562 and K562(HR3) cells with chromosomally integrated recombination substrate HR-EGFP/3′EGFP (3) were cultivated in RPMI1640 medium (Gibco, Waltham, MA, USA) with the addition of 15% FBS and 1.3% antibiotics and 10% FBS (Pan Biotech, Aidenbach, Germany), and 1% antibiotics, namely Penicillin-Streptomycin-Glutamine (Gibco, Waltham, MA, USA), respectively. All cells were incubated at 37°C with 5% $CO_2$. Prior to all experiments cells were checked for mycoplasma and contamination was excluded. K562 and K562(HR3) cell lines were authenticated by Microsynth AG, Balgach, Switzerland, using STR profiling.

### Plasmids and electroporation

Mammalian expression plasmids were designed with benchling.com for expression of the different *TP53* variants (see Table 1) and cloned by Origene (Rockville, MD, USA). Accordingly, *TP53* WT cDNA (NM_000546) with the precise nucleotide exchange identified in the GC-HBOC was cloned into the pCMV6-AN-Myc-DDK backbone. This construct was used for the design of all further variants. Plasmids were amplified using the Xtra Maxi NucleoBond from Macherey-Nagel (Düren, Germany). After cloning and after plasmid amplification each cDNA sequence was verified by sequencing (Microsynth AG, Balgach, Switzerland). Expression plasmid p5bPuroCMV-wtEGFP for WT EGFP was previously described (3).

Cells were transfected with 10 µg of expression plasmid for flow cytometric determination of recombination frequencies, for Western blotting as well as for DNA fiber assays via electroporation using GenePulser Xcell (BioRad, München, Germany). For individual determination of transfection efficiencies 10 µg of WT EGFP plasmid were added to the *TP53* variant expression plasmid in additional duplicates per experiment. For co-expression analysis 5 µg plasmid DNA for expression of the different *TP53* variants were mixed with 5 µg of *TP53* WT plasmid. After electroporation, cells were incubated in RPMI1640 (Gibco, Waltham, MA, USA) without antibiotics. Quality controlled stocks of plasmids sufficient for all rounds of functional analyses were generated for consistent test conditions.

**Recombination measurements**

K562(HR3) reporter cells with chromosomally integrated recombination substrate HR-EGFP/3′EGFP (3) were electroporated with expression plasmid for each *TP53* variant. Experiments were run in batches of randomly assembled VUS, whereby each batch was accompanied by internal references, i. e. *TP53* WT, empty vector without and with myc-tag 1:1 (EV), variants p.R110Pfs*, p.R213X and p.V31I, p.G360A representing P and B variants, respectively. Recombination frequencies were measured 72 h after electroporation by fluorescence-activated cell sorting (FACS) analysis using a CytoFLEX B3-R1-V0 flow cytometer with APD detectors and GFP-oD1 bandpass filter (Beckmann Coulter, Brea, CA, USA). Using the side scatter (SSC) versus forward scatter (FSC) plot, living cells were identified. Using the autofluorescence phycoerythrin (PE) versus GFP-oD1 plot, enhanced green fluorescent protein-positive (EGFP+) cells were identified. EGFP+ events were recorded in $1x10^6$ living cells/sample with triplicates/experiment. Mean recombination frequencies of WT expressing cells were set to one for each experiment (absolute mean frequency: $3x10^{-5}$). To test possible dominant-negative effects (DNEs), cells were co-electroporated with expression plasmid for the *TP53* variant (5 µg) and *TP53* WT (5 µg). Co-electroporation of EV and WT served as reference for LOF.

**Western Blotting**

Electroporated K562 cells were cultivated for 48 h. Proteins were extracted on ice in the cold for 30 min with a deep chromatin buffer (20 mM Tris-HCl, pH 8.0, 300 mM NaCL 0.5% NP-40, 0.5 mM EDTA, 10 mM NaF, protease inhibitor cocktail from Roche, Basel, Switzerland), which was followed by centrifugation for 15 min (15115xg). Proteins in the supernatant were electrophoretically separated and blotted on an Immobilon-P Membrane (PVDF) from Merck, Darmstadt, Germany. To ensure successful transfer the membranes were incubated in Ponceau Red (Sigma-Aldrich, St. Louis, MO, USA) and overnight with the following primary antibodies: anti-p53 (554293, BD, Franklin Lakes, NJ, USA), anti-p21 (556430, BD, Franklin Lakes, NJ, USA), anti-Vinculin (sc-73614, Santa Cruz, Dallas, TX, USA), anti-Ku70 (ab202022, Abcam, Eugene, OR,USA ), anti-Actin (B0719, Santa Cruz, Dallas, TX, USA). HRP anti-mouse (H+L) and HRP anti-rabbit (H+L) (Rockland, Pottstown, USA) were used as secondary antibodies. Chemiluminescence detection was done using ChemidocMP System (Bio-Rad, Munich, Germany). The protein levels were quantified in the linear range by ImageLab Software (Bio-Rad, Munich, Germany), corrected with the values of the loading control and normalized to the p53 level in the *TP53* WT control.

**DNA fiber spreading assay**

Newly synthesized DNA was labelled in 20 min pulses, first with 20 µM CldU (5-chloro-2-deoxyuridine, Cayman Chemicals, Ann Arbor, MI, USA) and second with 200 µM IdU (5-iodo-2-deoxyuridine, Sigma-Aldrich, St. Louis, MO, USA). Between the pulses, K562 cells were spun down at 1030xg for 2 min. After the IdU pulse cells were spun down at 1030xg for 2 min and washed with cold PBS (Gibco, Waltham, MA, USA). For the fiber spreading 2800 cells were transferred to a glass slide and gently mixed with 6 µl lysis buffer consisting of 0.5 % SDS (Merck, Darmstadt, Germany), 200 mM Tris-HCl, pH 7.4 (Sigma-Aldrich, St.Louis, MO, USA), 50 mM EDTA (Roth, Karlsruhe, Germany). Immediately after lysis cells were spread in a spiral shape. Slides were tilted to ensure further spreading and dried. The DNA was fixed for 5 min in 3:1 Methanol (Merck, Darmstadt, Germany) and Acetic Acid (VWR, Radnor, PA, USA).

Then, the slides were dried at RT and stored in 70% ethanol at 4°C overnight. Next, slides were incubated in 100% Methanol (Merck, Darmstadt, Germany) for 5 min. For denaturation of the DNA, cells were incubated in 2.5 N HCl for 1 h. PBS containing 5% BSA (Sigma Aldrich, St. Louis, MO, USA) was used to block for 45 min at 37 °C. Immunofluorescence staining was performed by adding primary antibody mix of Ms anti-BrdU (BD Biosciences, Franklin Lakes, NJ, USA) and Rt anti-BrdU (Abcam, Cambridge, United Kingdom) in PBS with 0.5 % BSA for 1 h at RT. After washing with 0.05% Tween 20 (Gibco, Waltham, MA, USA; Roth, Karlsruhe, Germany) in PBS (PBST) and PBS the slides were incubated with the secondary antibody mix with Gt anti-Ms Alexa 555 and Dk anti-Rt Alexa 488 (Invitrogen, Carlsbad, CA, USA) for 1 h at RT. Slides were washed again multiple times with PBST and PBS and covered with Dabco Mowiol mixture (Sigma-Aldrich St. Louis, MO, USA; Merck, Darmstadt, Germany). The slides were analyzed with the fluorescence microscope Keyence BZ-9000 (Keyence, Neu-Isenburg, Germany) and track lengths measured with Fiji/Image J (NIH and Laboratory for Optical and Computational Instrumentation (LOCI) University of Wisconsin, WI, USA) using the same computer screen throughout the experimental series. Images were taken in the region of the upper spiral, with maximum separation and validated integrity of the individual DNA fibers (4).

**Proximity ligation assay (PLA).**

PLA was carried out according to the manufacturer's instruction (Sigma-Aldrich, St. Louis, MO, USA). Briefly, transfected K562 cells were cultivated for 48 h and treated with Mitomycin C (Sigma-Aldrich, St. Louis, MO, USA) for 45 min. Subsequently, cells were released in fresh culture medium for 3 h. Then, 75000 cells were spun onto a coated slide and fixed with 3.7% formaldehyde in PBS for 10 min. PLA staining was carried out according to manufacturer's protocol (Sigma-Aldrich, St. Louis, MO, USA). Hereby cells were double-labeled with the primary antibodies anti-DNA Polymerase ι (mouse, mAb, OTI3B5, ThermoFisher #MA5-26503) and anti-p53pSer15 (rabbit, polyclonal, Cell Signalling #9284) before PLA-staining was performed with Duolink In Situ PLA probe Anti-Rabbit PLUS (DUO92002) and Duolink In Situ PLA probe Anti-Mouse MINUS (DUO92004). Duolink In Situ Detection Reagents Green

(DUO92014) and Orange (DUO92007) were used for ligation, amplification and detection. The slides were covered with Duolink In Situ Mounting Media with DAPI (DUO82040, Sigma-Aldrich, St. Louis, MO, USA). Microscopic imaging was performed using a Zeiss fluorescence microscope (Carl Zeiss AG, Oberkochen, Germany) and analyzed using ZEN Blue software (Carl Zeiss AG) with consistent settings maintained across all samples to ensure reproducibility.

## Supplementary References


1. Hauke J, Horvath J, Groß E, Gehrig A, Honisch E, Hackmann K et al. Gene panel testing of 5589 BRCA1/2-negative index patients with breast cancer in a routine diagnostic setting: results of the German Consortium for Hereditary Breast and Ovarian Cancer. Cancer Med 2018; 7(4):1349–58.

2. Rhiem K, Engel C, Graeser M, Zachariae S, Kast K, Kiechle M et al. The risk of contralateral breast cancer in patients from BRCA1/2 negative high risk families as compared to patients from BRCA1 or BRCA2 positive families: a retrospective cohort study. Breast Cancer Res 2012; 14(6):R156.

3. Akyüz N, Boehden GS, Süsse S, Rimek A, Preuss U, Scheidtmann K-H et al. DNA substrate dependence of p53-mediated regulation of double-strand break repair. Mol Cell Biol 2002; 22(17):6306–17.

4. Castaño BA, Schorer S, Guo Y, Calzetta NL, Gottifredi V, Wiesmüller L et al. The levels of p53 govern the hierarchy of DNA damage tolerance pathway usage. Nucleic Acids Res 2024; 52(7):3740–60.

5. Kato S, Han S-Y, Liu W, Otsuka K, Shibata H, Kanamaru R et al. Understanding the function-structure and function-mutation relationships of p53 tumor suppressor protein by high-resolution missense mutation analysis. Proc Natl Acad Sci U S A 2003; 100(14):8424–9.

6. Kotler E, Shani O, Goldfeld G, Lotan-Pompan M, Tarcic O, Gershoni A et al. A Systematic p53 Mutation Library Links Differential Functional Impact to Cancer Mutation Pattern and Evolutionary Conservation. Mol Cell 2018; 71(1):178-190.e8.

7. Giacomelli AO, Yang X, Lintner RE, McFarland JM, Duby M, Kim J et al. Mutational processes shape the landscape of TP53 mutations in human cancer. Nat Genet 2018; 50(10):1381–7.

8. Funk JS, Klimovich M, Drangenstein D, Pielhoop O, Hunold P, Borowek A et al. Deep CRISPR mutagenesis characterizes the functional diversity of TP53 mutations. Nat Genet 2025; 57(1):140–53.

9. Natan E, Baloglu C, Pagel K, Freund SMV, Morgner N, Robinson CV et al. Interaction of the p53 DNA-binding domain with its n-terminal extension modulates the stability of the p53 tetramer. J Mol Biol 2011; 409(3):358–68.

# Supplementary Figure 1

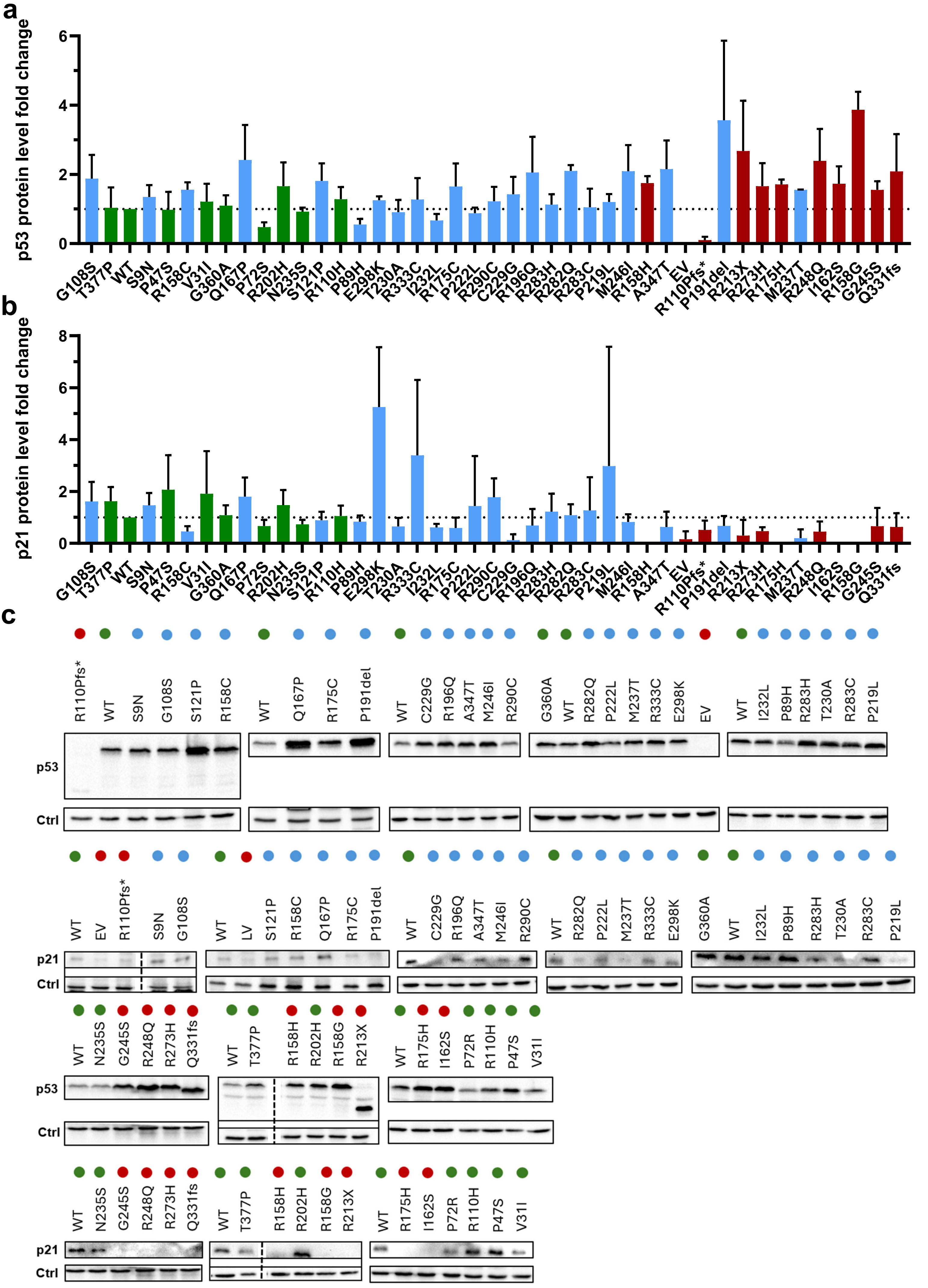

**Supplementary Figure 1. p53 and p21 protein expression.**
Cells were harvested 48 h after electroporation of K562 cells, proteins extracted with a deep chromatin buffer (Materials and Methods) and subjected to Western blotting. Columns represent mean values and SD of specific band intensities (p53, p21) corrected for protein loading by at least one control band intensity each (Actin, Ku70, Vinculin) and normalized as fold changes to the means of a *TP53* WT expressing sample from the same experimental day, immunoblot and exposure time. Such normalized expression levels of *TP53* VUS (blue), functional (green) and non-functional control variants (red) are presented in the order of the waterfall plot showing recombination data in Fig. 2c. N=2-24.
**a** Protein levels of p53.
**b** Protein levels of p21.
**c** Representative Western blots. Western blots visualize band intensities of p53, p21 and one of the loading controls (Ctrl) after expression of different *TP53* variants analyzed in this study. Colored dots label VUS (blue) and control variants (P/LP, red; B/LB, green). Frames encompass images from the same immunoblot and exposure time, dotted lines mark cropping within. For uncropped Western blots and all loading controls see Source Data 1.

## Supplementary Figure 2

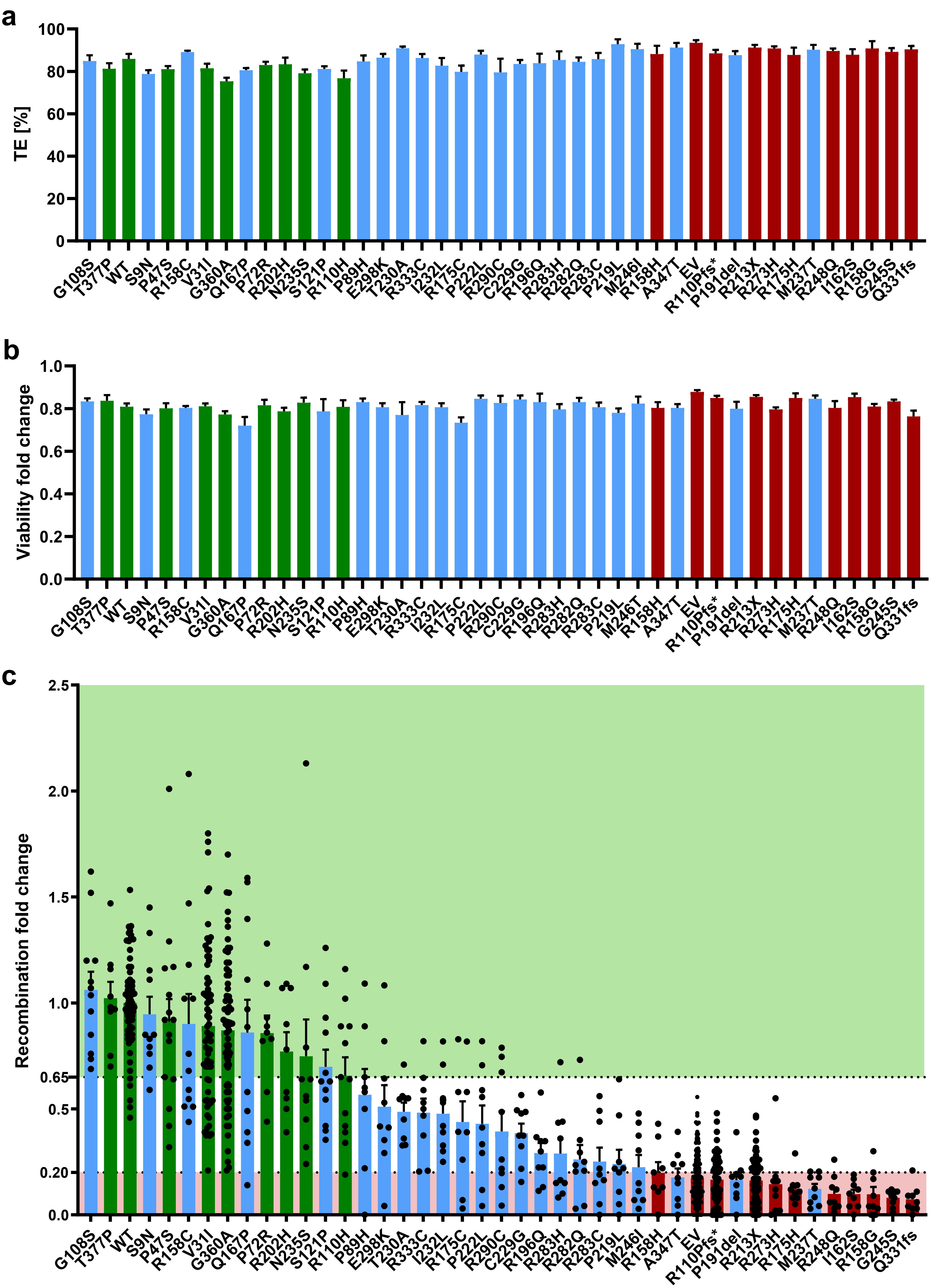

**Supplementary Figure 2. Quality controls for DNA recombination experiments.**
Samples for recombination measurements in Fig. 2c were additionally used for determination of viabilities in the SSC/FSC gate. *TP53* variant expressing samples for recombination measurements were accompanied by samples co-expressing EGFP for determination of transfection efficiencies (TE). These data are presented in the same order and color scheme as the waterfall plot in Fig. 2c.
**a** Transfection efficiency. The percentages (%) of K562(HR3) cells expressing EGFP 72 h after electroporation with *TP53* variant and EGFP expression plasmids are indicated. Columns represent mean values and SEM. N=2-5, n=4-10.
**b** Viability. Percentages of living cells in K562(HR3) samples 72 h after electroporation with *TP53* variant expression plasmids were identified in the SSC/FSC gate during flow cytometric recombination measurements and normalized to viabilities in non-transfected control cultures (fold change). Columns represent mean values and SEM. N=3-17, n=8-102.
**c** Individual recombination measurements. Recombination fold change for each individual sample and experiment after expression of each *TP53* variant is shown (for statistically significant differences see Fig. 2c). Mean values with SEM, represented by columns and bars, and individual values, represented by black dots. N=3-24, n=8-141.

## Supplementary Figure 3

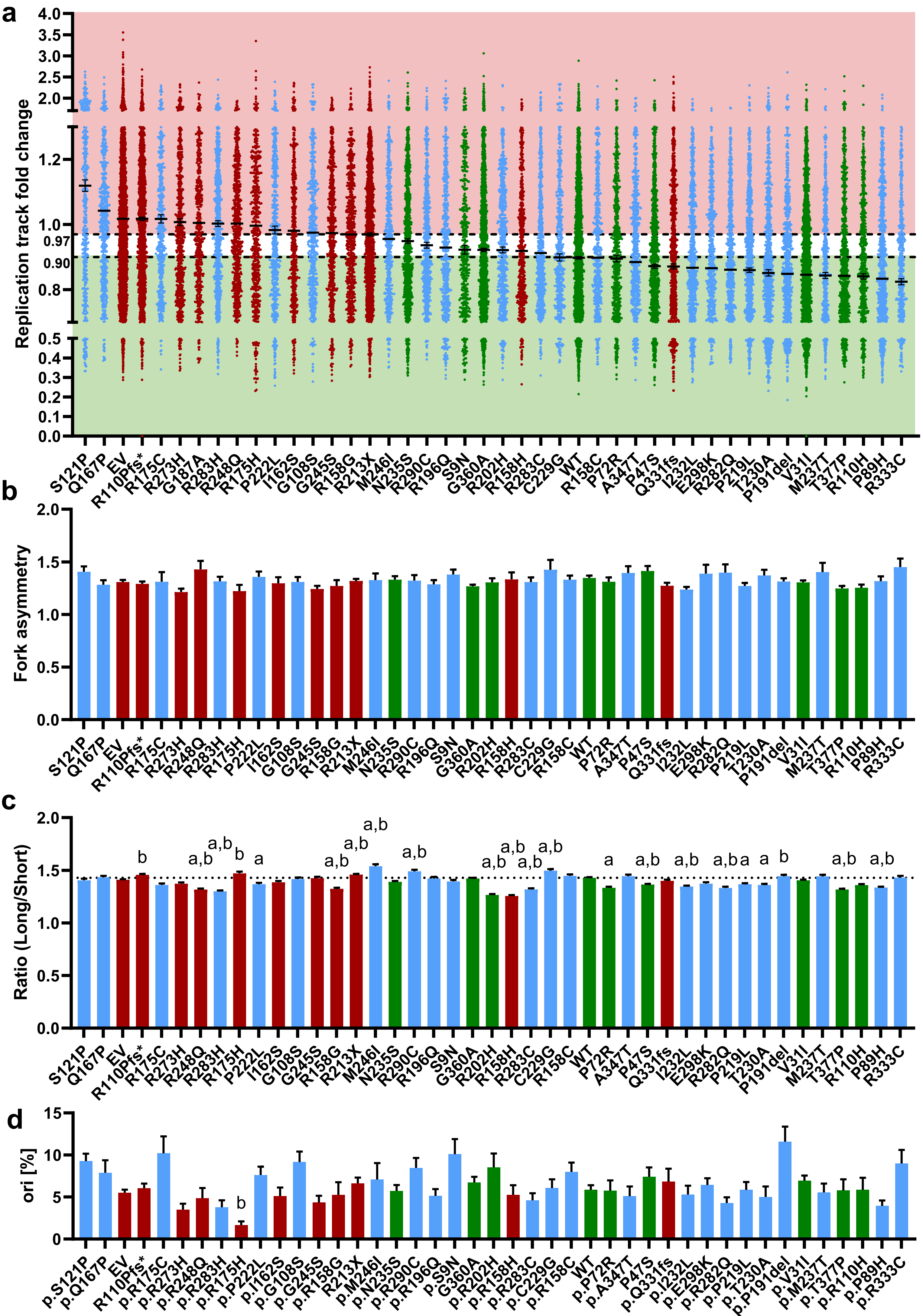

**Supplementary Figure 3. Individual DNA fiber track lengths, fork asymmetries and fiber track ratios.**

Samples for DNA fiber spreading assays in Fig. 3c were additionally used for assessment of fork stalling via quantification of asymmetries on tricolored forks and long/short ratios of sequential CldU and IdU tracks. Data for the different *TP53* variant expressing samples are presented in the same order and color code as in the waterfall plot in Fig. 3c. Statistically significant differences were calculated by use of Kruskal-Wallis H-test followed by the two-tailed Mann-Whitney U test. Statistically significant differences ($p<0.0001$) of the variant-specific values as compared to WT and EV are indicated by a and b, respectively. Precise p-values are listed in Supplementary Table 1.

**a** Individual fiber track lengths. Individual IdU track lengths from DNA fiber spreading assays normalized to the means from p.R110Pfs* and p.R213X expressing samples from the same experimental day are displayed as a bee swarm graph. Means with SEM are indicated (N=3-14, n=635-6677).

**b** Fork asymmetries. Columns represent the mean values with SEM (N=3-14, n=15-326).

**c** Fiber track ratio. Columns represent the mean values with SEM (N=3-14, n=635-6677).

**d** Percentage of 1st pulse origins within all countable replication structures. Columns represent the mean values with SEM (N=3-14; n=7-113).

# Supplementary Figure 4

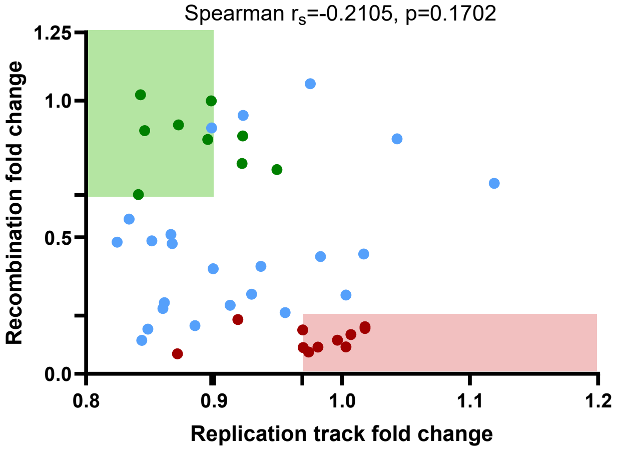


**Supplementary Figure 4. Analyses of correlations between variant-specific recombination and replication track data.**

Spearman correlation analyses were performed comparing variant-specific mean recombination and replication fold changes as listed in Table 1 and graphically displayed in Fig. 2c and Fig. 3c. Green and red areas mark functional and non-functional activity ranges, as defined in Fig. 2c and Fig. 3c, respectively.

# Supplementary Figure 5

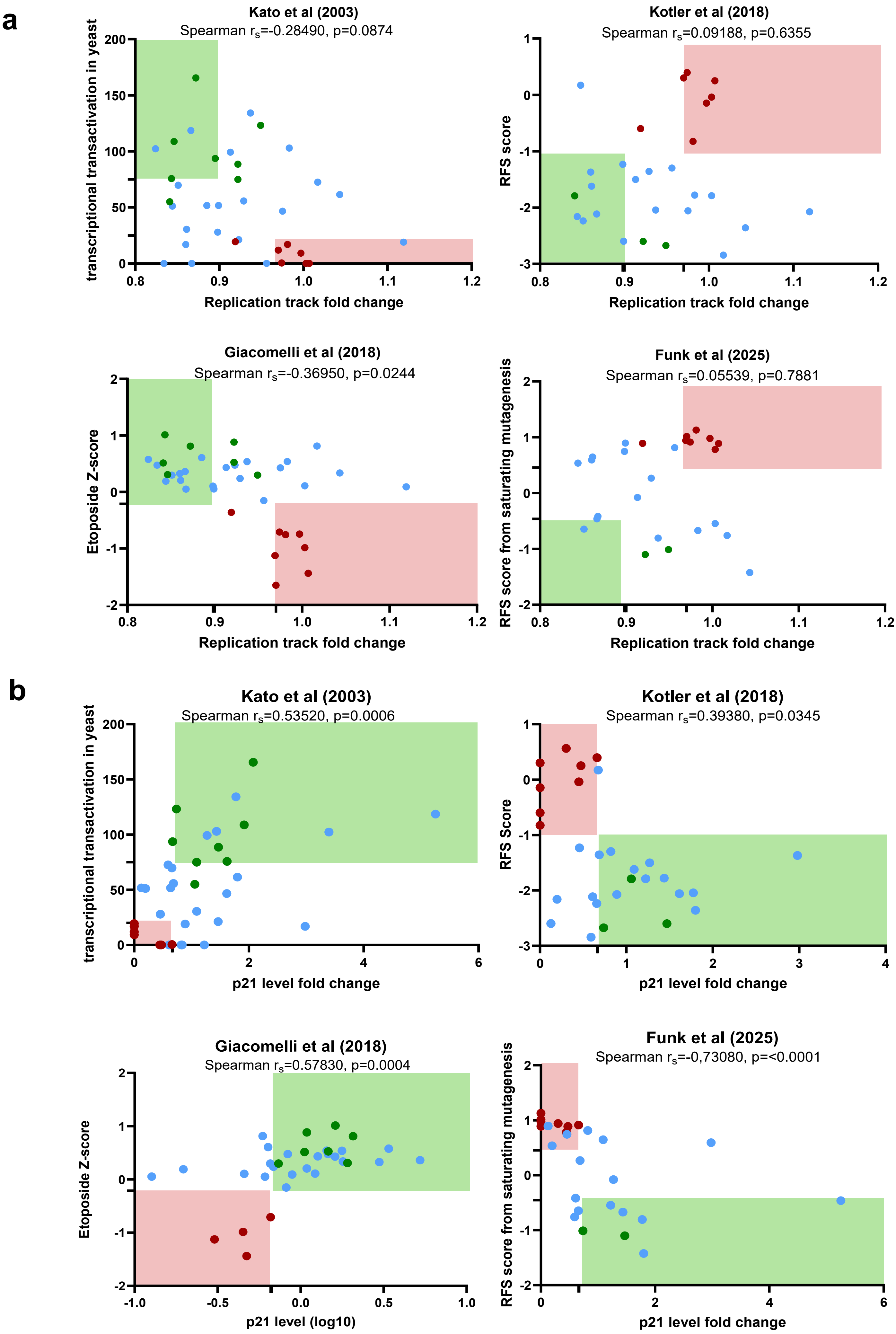

**Supplementary Figure 5. Analyses of correlations between replication track changes or p21 levels and data from assays based on canonical p53 functions.**

Spearman correlation analyses were performed comparing mean IdU replication track lengths (**a**) or p21 protein levels (**b**) listed in Table 1 and graphically displayed in Fig. 3c and Supplementary Fig. 1b with TA in yeast (5), with RFS from human H1299 cells (6), with etoposide Z-score from human A549 cells (7) and with RFS from human HCT116 cells (8). p21 levels were transformed to log10 in the correlation analyses with data from Giacomelli *et al.* (7).

# Supplementary Figure 6

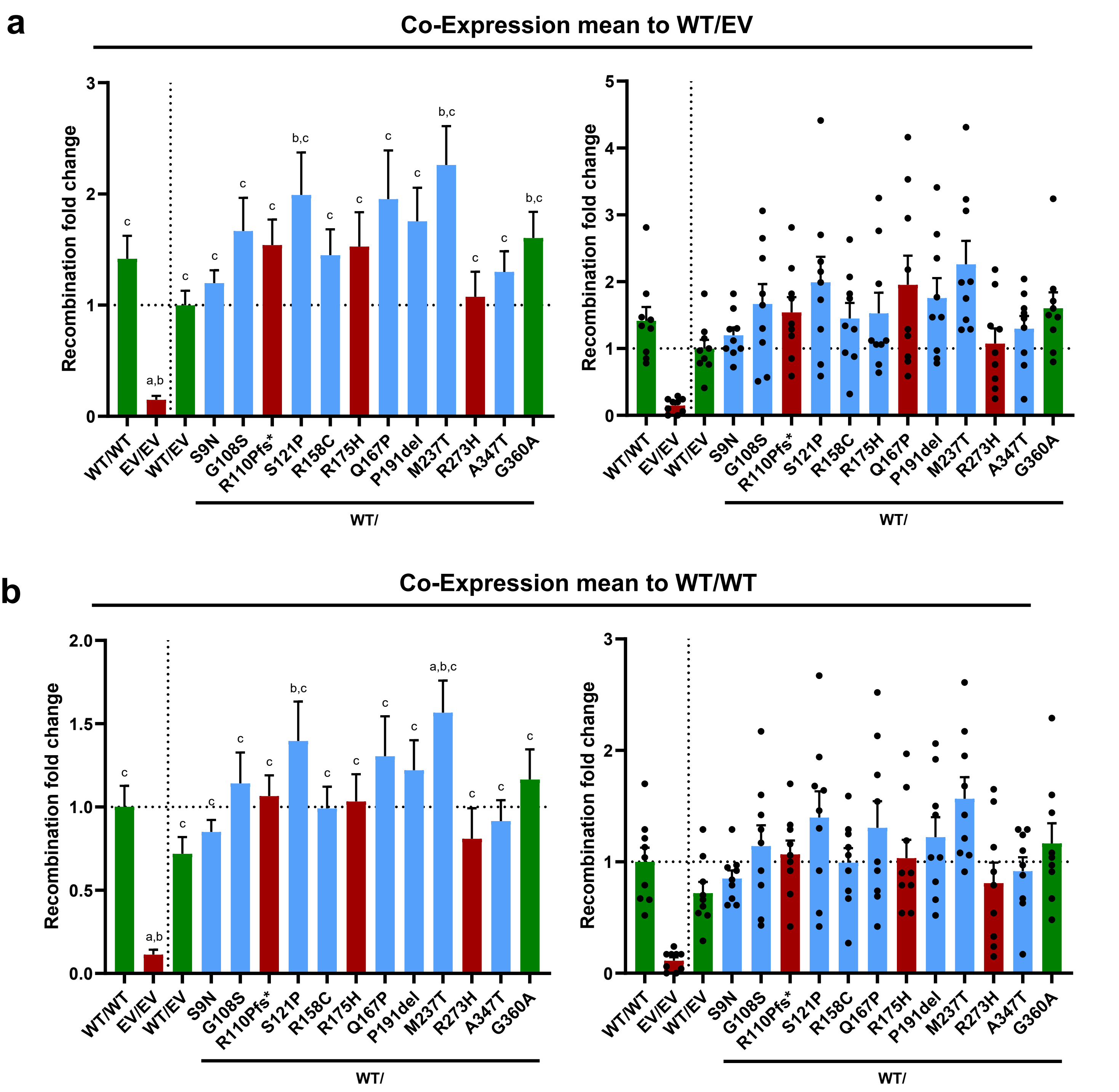


**Supplementary Figure 6. Recombination as a function of *TP53* variant co-expressed with WT.**

Recombination measurements were performed as in Fig. 2 except for co-electroporation of expression plasmids for *TP53* WT (5 µg) and each selected variant (5 µg). Data are plotted for P/LP variants (red), B/LB variants (green) or VUS (blue) according to the position along the aa sequence of the p53 protein. Controls were electroporated with 10 µg WT (WT/WT), 10 µg EV (EV/EV) or 5 µg WT plus 5 µg EV (WT/EV). Recombination frequencies after co-expression of *TP53* WT (WT/) and the indicated variants were individually normalized to WT/WT values. Columns represent mean values with SEM. N=3, n=9.

Co-expression of *TP53* WT and different variants normalized to WT/EV (**a**) and WT/WT (**b**). Statistical analyses were performed by use of Kruskal-Wallis H test followed by Mann-Whitney U test, two-sided. Statistically significant differences of the means specific for selected variants tested in co-expression samples versus WT/WT (a), WT/EV (b) and EV/EV (c) are shown. Specific p-values can be found in Supplementary Table 1. Individual recombination frequencies after co-expression of *TP53* WT and the indicated variants. Individual values are visualized by black dots.

# Supplementary Figure 7

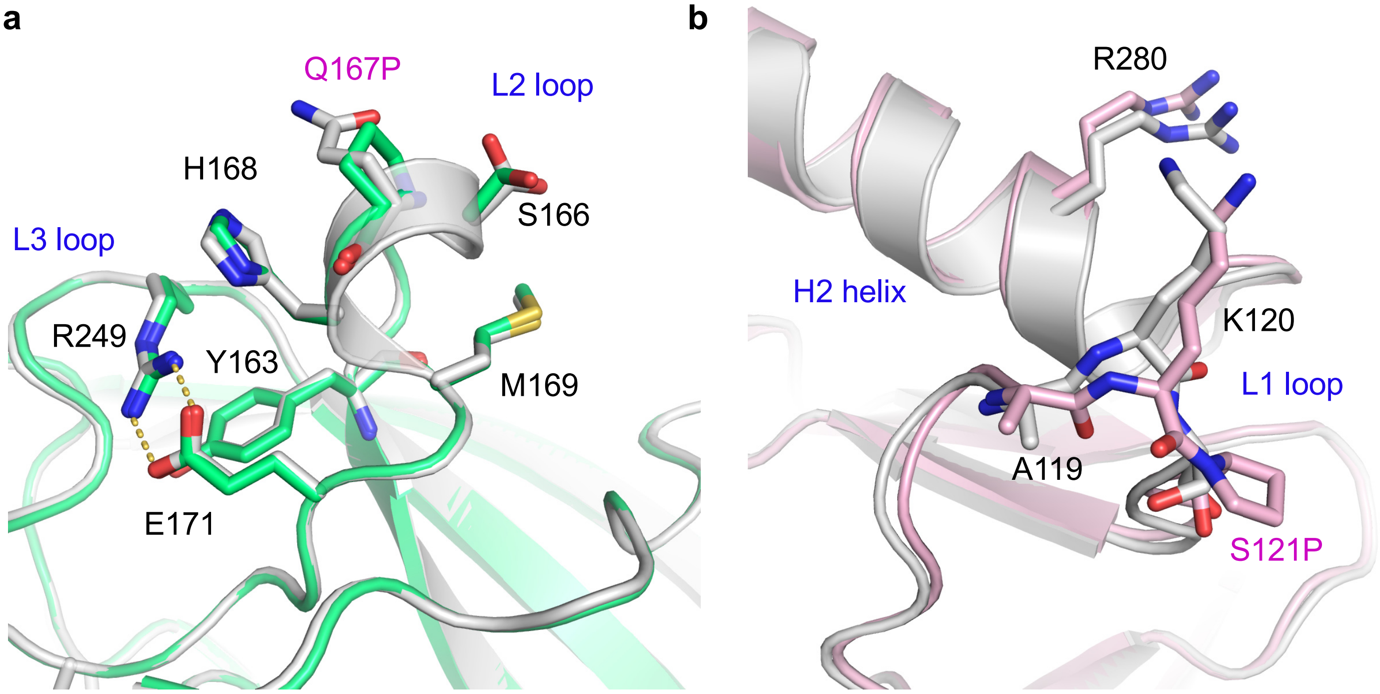


**Supplementary Figure 7. Structural effects of variants p.S121P and p.Q167P in the DBD.**
**a** Close-up view of the mutation site in the p.Q167P variant AlphaFold model (green) superimposed onto the wild-type DBD (gray; PDB entry 2XWR) (9). The salt bridge between E171 and R249 is shown as a yellow dashed line.
**b** Close-up view of the mutation site in the p.S121P variant AlphaFold model (pink) superimposed onto the WT DBD (gray; PDB entry 2XWR). Selected residues are highlighted as stick models.
