# Supplementary material for: Evaluation of non-canonical p53 functions in DNA replication and recombination for variant classification
Source: Cell Death Dis. 2026 Feb 28;17(1):292. doi: 10.1038/s41419-026-08463-0 (PMC13031496; doi:10.1038/s41419-026-08463-0)

Ad Supplementary Fig. 1c: Protein level of p53: TP53 VUS

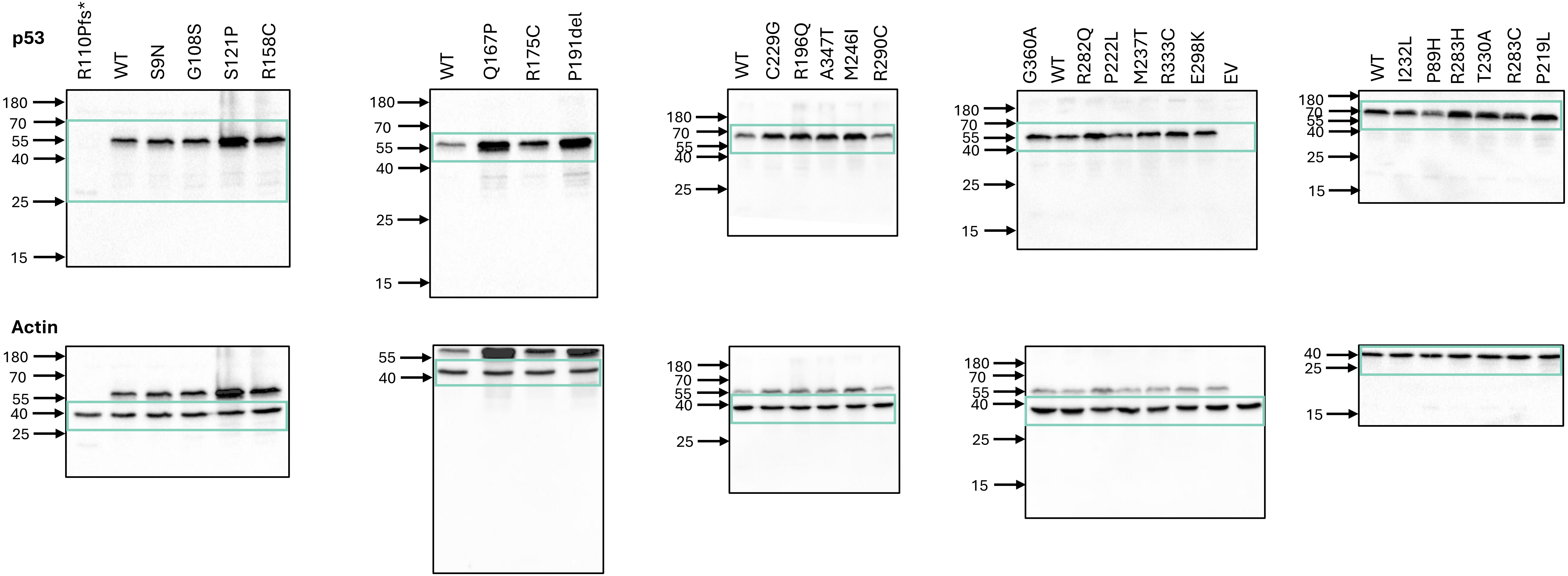

Ad Supplementary Fig. 1c: Protein level of p53: TP53 control variants

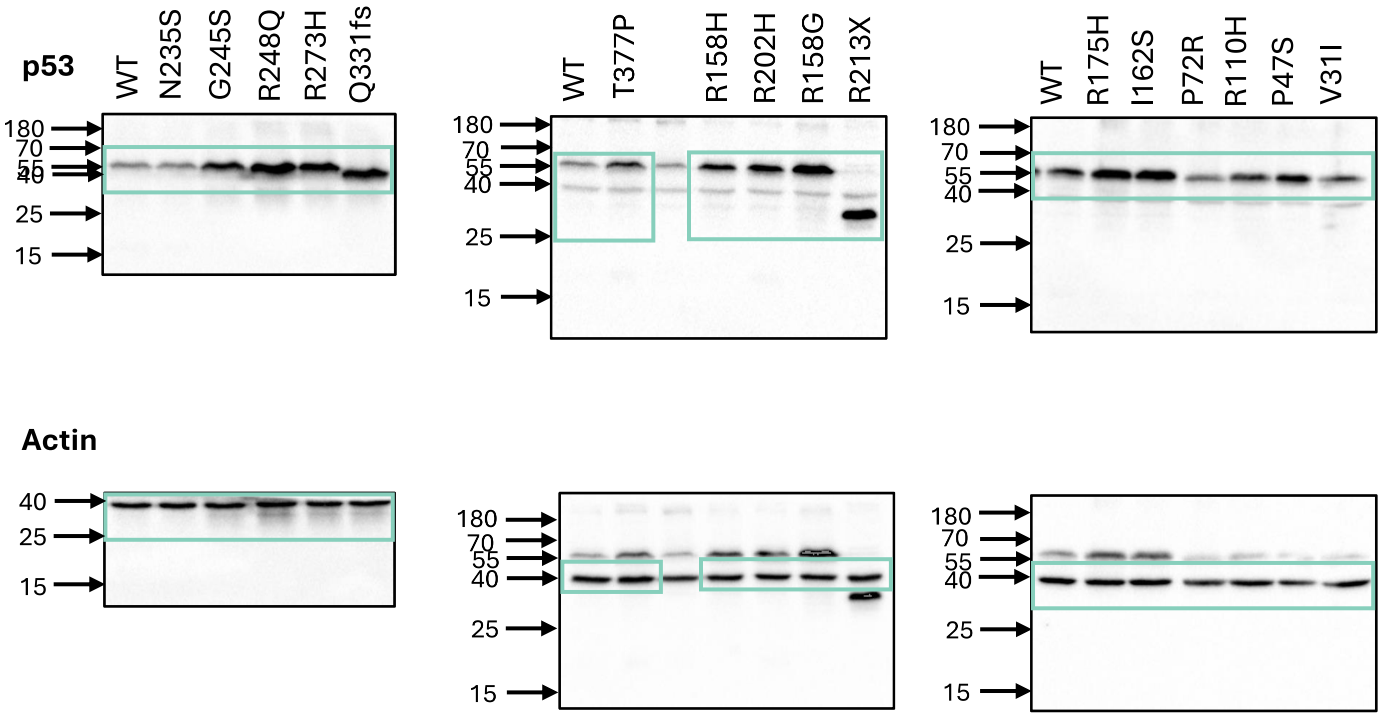

Ad Supplementary Fig. 1c: Protein level of p21: TP53 VUS

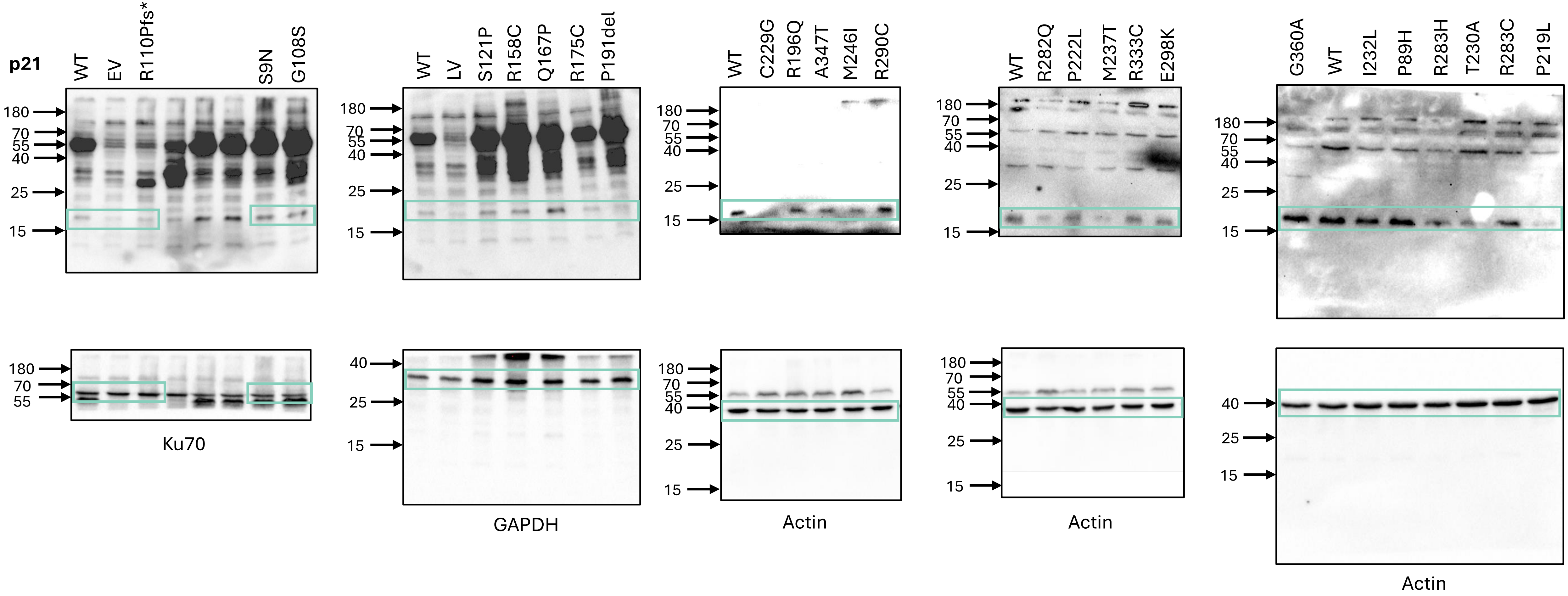

Ad Supplementary Fig. 1c: Protein level of p21: TP53 control variants

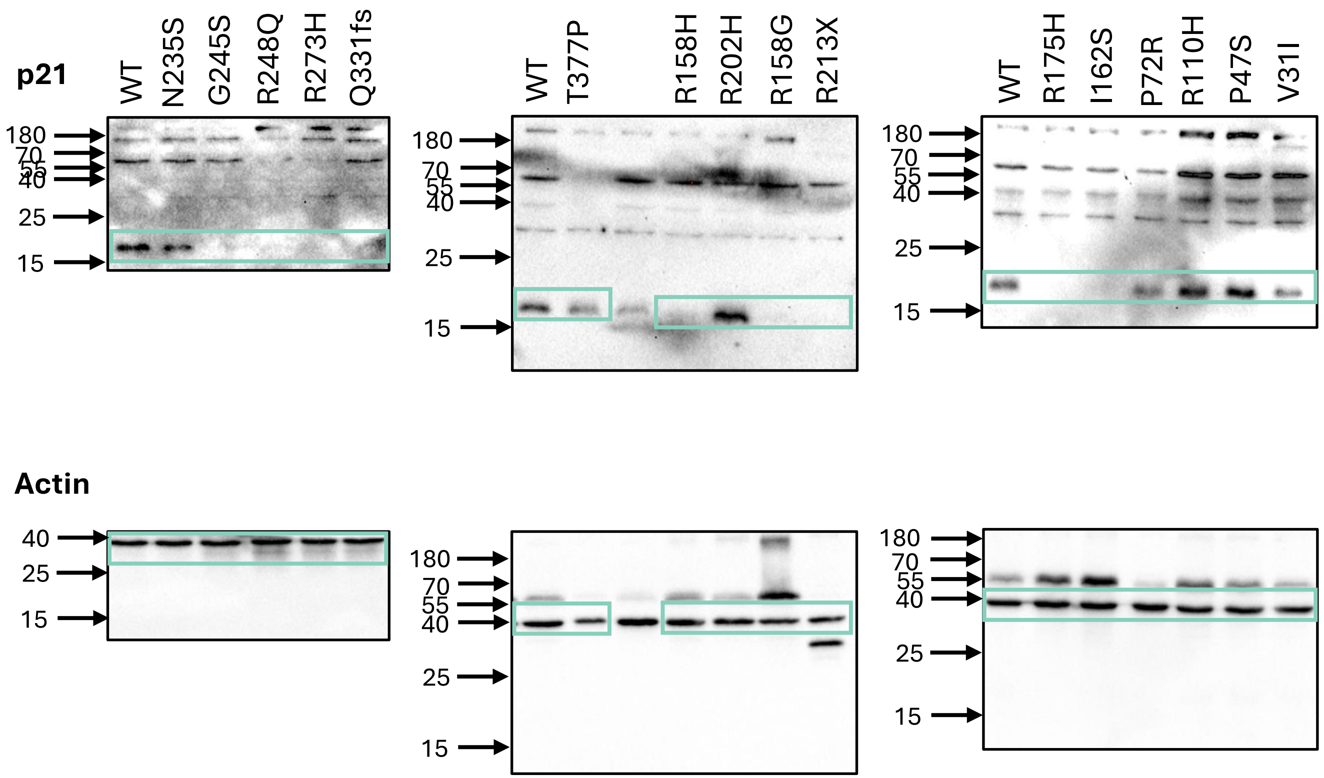

Supplement: Supplementary file 3 — Source Data [file 41419_2026_8463_MOESM3_ESM.pdf]
